# Roadmap on biology in time varying environments

**DOI:** 10.1088/1478-3975/abde8d

**Published:** 2021-05-17

**Authors:** Arvind Murugan, Kabir Husain, Michael J Rust, Chelsea Hepler, Joseph Bass, Julian M J Pietsch, Peter S Swain, Siddhartha G Jena, Jared E Toettcher, Arup K Chakraborty, Kayla G Sprenger, T Mora, A M Walczak, O Rivoire, Shenshen Wang, Kevin B Wood, Antun Skanata, Edo Kussell, Rama Ranganathan, Hong-Yan Shih, Nigel Goldenfeld

**Affiliations:** 1 James Franck Institute, Department of Physics, University of Chicago, Chicago, IL 60637, United States of America; 2 Department of Molecular Genetics and Cell Biology, University of Chicago, Chicago, IL 60637, United States of America; 3 Department of Physics, University of Chicago, Chicago, IL 60637, United States of America; 4 Department of Medicine, Feinberg School of Medicine, Division of Endocrinology, Metabolism and Molecular Medicine, Northwestern University, Chicago, IL 60611, United States of America; 5 SynthSys: Centre for Synthetic and Systems Biology, School of Biological Sciences, University of Edinburgh, Edinburgh EH9 3BF, United Kingdom; 6 Department of Molecular Biology, Princeton University, Princeton, NJ 08544, United States of America; 7 Department of Chemical Engineering, Massachusetts Institute of Technology, Cambridge, MA 02139, United States of America; 8 Department of Physics, Massachusetts Institute of Technology, Cambridge, MA 02139, United States of America; 9 Department of Chemistry, Massachusetts Institute of Technology, Cambridge, MA 02139, United States of America; 10 Institute for Medical Engineering & Science, Massachusetts Institute of Technology, Cambridge, MA 02139, United States of America; 11 Ragon Institute of the Massachusetts General Hospital, Massachusetts Institute of Technology, and Harvard, Cambridge, MA 02139, United States of America; 12 Laboratoire de physique, Ecole normale supérieure, CNRS, PSL Research University, Paris, France; 13 Center for Interdisciplinary Research in Biology (CIRB), Collège de France, CNRS, INSERM, PSL Research University, Paris, France; 14 Department of Physics and Astronomy, University of California, Los Angeles, Los Angeles, CA 90095, United States of America; 15 Departments of Biophysics and Physics, University of Michigan, Ann Arbor, MI 48109-1055, United States of America; 16 Center for Genomics and Systems Biology, New York University, 12 Waverly Place, Rm. 206, New York, NY 10003, United States of America; 17 Center for Physics of Evolving Systems, Biochemistry & Molecular Biology, and the Pritzker School for Molecular Engineering, University of Chicago, Chicago IL 60637, United States of America; 18 Department of Physics, University of Illinois at Urbana-Champaign, Champaign, Illinois 61801, United States of America; 19 Institute of Physics, Academia Sinica, Taipei 11529, Taiwan; 20 Carl R Woese Institute for Genomic Biology, University of Illinois at Urbana-Champaign, Champaign, Illinois 61801, United States of America

**Keywords:** allostery, time varying environments, cellular signaling, evolution, environmental dynamics, signaling dynamics

## Abstract

Biological organisms experience constantly changing environments, from sudden changes in physiology brought about by feeding, to the regular rising and setting of the Sun, to ecological changes over evolutionary timescales. Living organisms have evolved to thrive in this changing world but the general principles by which organisms shape and are shaped by time varying environments remain elusive. Our understanding is particularly poor in the intermediate regime with no separation of timescales, where the environment changes on the same timescale as the physiological or evolutionary response. Experiments to systematically characterize the response to dynamic environments are challenging since such environments are inherently high dimensional. This roadmap deals with the unique role played by time varying environments in biological phenomena across scales, from physiology to evolution, seeking to emphasize the commonalities and the challenges faced in this emerging area of research.

## 1. Introduction

The natural world is ever changing, and living organisms evolve to thrive in these changing circumstances. Available sugars change over the course of a bacterium’s lifetime, pathogens seen by our immune systems change with the seasons, and an organism’s ecological niche can change over evolutionary timescales. Biological organisms have mechanisms to respond to such dynamical environments on all these scales. However, the response to dynamical environments has been hard to study in a systematic manner since the space of dynamical environments is inherently high dimensional, requiring high-throughput time-resolved measurements. What are the outstanding challenges and opportunities in studying such dynamic phenomena? In this roadmap, we present perspectives from such diverse fields as evolution and ecology, cellular signaling, development, and circadian biology.

Biology in time-varying environments has been mostly studied when there is a separation of timescales between environmental dynamics and the biological response. Such dynamics fall into two limits that we can understand using effective static frameworks. Slow changes in the environment elicit a ‘quasi-static’ response that is well-adapted to the effectively static environment at that moment in time. Conversely, rapid environmental variations faster than biological response timescales are filtered out, and enter only as enhanced fluctuations around a static backdrop.

In contrast to such fast or slow variations, if the environment changes on an intermediate timescale—that is, on timescales comparable to the biological response—adaptation to one environmental condition is only partially complete when adaptation to another condition must begin. The dynamics of the environment thereby couple to biological transients, and adapting organisms must reckon with both changes in their surroundings as well as the precise time over which they occur.

In this roadmap, we present perspectives from diverse fields of biology, highlighting how probing biological systems at these intermediate timescales can elicit novel, history-dependent responses from cells, organisms, and populations. These include regular environmental rhythms, such as the 24 h day–night cycle. Rust explores how the need to reliably process temporal information constrains the architecture of biological circuits, while Hepler and Bass describe the delicate coupling between metabolic and circadian cycles that underlie health and disease. Other temporal cues may be less predictable, such as the intermittent onset of extracellular stresses. New technologies are required to probe cellular responses to these signals. Pietsch and Swain describe how microfluidics, combined with single cell imaging, can be used to study the encoding of extracellular stimuli in intracellular dynamics, while Jena and Toettcher draw lessons from optogenetic studies of the metazoan Ras/Erk system to outline the role of signaling dynamics in homeostasis and development.

Time varying environments can also be exogenously applied as a control strategy, directing biological processes to desirable outcomes. Chakraborty and Sprenger describe temporal vaccination protocols that direct the immune system toward broadly neutralising antibodies (Abs) that can bind multiple antigens. In contrast, Wood describes how time-varying doses of antibiotics can slow the evolution of microbes resistant to multiple drugs. Mora, Walczak, and Rivoire situate these protocols in classic strategies for bet-hedging, while Wang describes how immune systems learn from, generalise, and affect a changing pathogenic environment.

Time-varying environments on longer timescales can shape evolutionary processes. Skanata and Kussell describe the evolution of regulated cellular growth as a response to fluctuating environments seen over evolutionary history. Further, time-varying environments seen in the past may imprint themselves physically into the products of evolution, as described by Ranganathan for the evolution of allostery in proteins. Finally, Shih and Goldenfeld take into consideration that time-varying environments are often not set externally by abiotic factors but by other coevolving species, giving rise to a rich set of self-tuned, eco-evolutionary dynamics.

## 2. Biological oscillators as signal processing devices

### 2.1. Status

Most laboratory experiments in biology involve cells or organisms in culture living either in constant conditions, or in conditions that have a simple time-varying structure. In contrast, the natural environments under which these organisms evolved have rhythms and fluctuations on many timescales. Some of this temporal structure corresponds to signals that are potentially actionable in the sense that they carry predictive information about the future. Familiar examples include the daily rhythm of day and night, the annual rhythm of seasons, and the diurnal, semidiurnal, and monthly rhythms of the tides. Beyond these astrophysical rhythms, many microbes may live in environments with varying degrees of regularity. For example, bacteria living in the human gut may experience pulsatile nutrient rhythms corresponding to human mealtimes.

**Figure 1.**
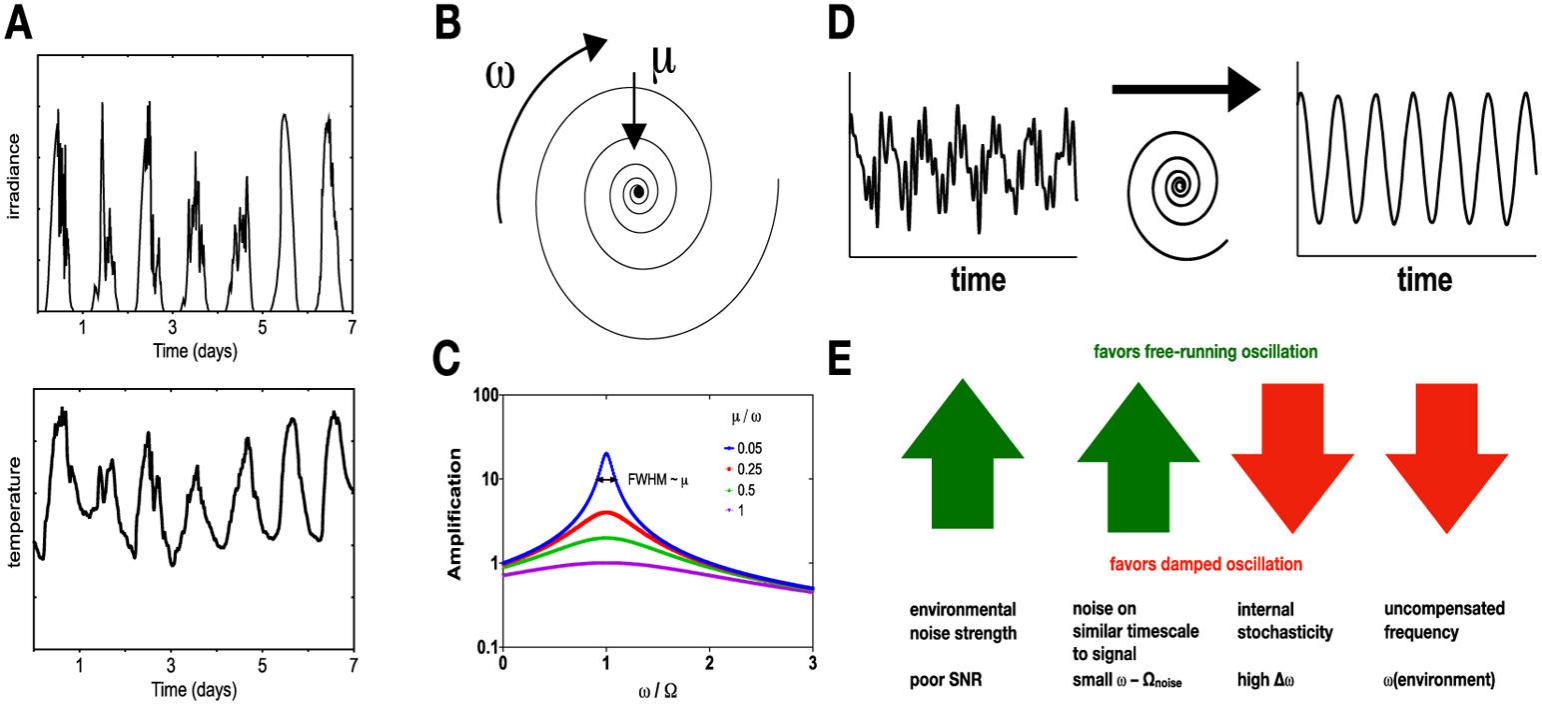
Signal processing by spiral node dynamics. (A) Cartoon of day–night cycles in irradiance (top) and temperature (bottom). (B) Phase plane dynamics of a symmetric spiral node. *ω* characterizes the angular frequency, *μ* characterizes the rate of decay. (C) Signal amplification by linear oscillator as a function of the relative mismatch between the natural frequency *ω* and the signal frequency Ω. (D) Simulated example of a regular rhythm heavily contaminated by noise at many different frequencies proposed by a spiral node network. (E) Environmental and internal factors predicted to favor increasing or decreasing the damping biological oscillators.

These potentially predictive signals are usually contaminated with noise, irrelevant fluctuations that do not contain usable information. For any particular case, noise will be strongest at different parts of the frequency spectrum. As an example, consider the obvious day–night rhythm in illumination. This signal is partially obscured by higher frequency fluctuations due to cloud cover etc. Temperature also exhibits daily cycles, but these rhythms are more prominently obscured by low frequency fluctuations due to longer timescale change in the weather (figure 1(A)).

### 2.2. Current and future challenges

These observations naturally raise the question of which strategies living systems might use to deal with the challenge of perceiving an informative signal in the presence of irrelevant noise. In the case of daily cycles, two general behaviors have been observed experimentally. The first is physiology that responds rhythmically to daily rhythms in the environment, but these rhythms fade out when the environment is held constant. Damped daily rhythms have been reported in many organisms, especially microbes, including budding yeast [2], pseudomonads [9], purple bacteria [4], and the cyanobacterial clade *Prochlorococcus* [3]. An alternative is rhythmic physiology that is self-sustaining in constant conditions, known as a circadian rhythm, the name referring to internally generated rhythm that is about a day in period. Most animals and plants, some fungi, and many cyanobacteria show self-sustaining rhythms. These timing mechanisms are in general implemented by elaborate biochemical circuitry, and as the details may vary markedly from species to species, it may be impossible to make truly general statements about molecular mechanism.

### 2.3. Advances in science and technology to meet challenges

To attempt to make generic statements about physiology in time-varying environments, we turn to an idea from the study of dynamical systems called normal form theory. The idea is that, sufficiently close to a fixed point of the system, many features of the system can be approximately understood in terms of a low-dimensional description that is universal, in the sense that almost all systems with the same qualitative behavior should follow equivalent equations. As a first step, consider the behavior of a system, such as an hourglass mechanism, which decays to a stable steady state when the environment is constant. Sufficiently close to the steady state, the dynamics can treated by a linear approximation. In general, the eigenvalues of the resulting linear system with the least negative real part (and hence slowest decaying) will be a complex conjugate pair. This results in the normal form for a spiral node (figure 1(B)):

$$\frac{d}{dt}\left(\begin{array}{c} x\\ y \end{array}\right)=\left(\begin{array}{cc} \mu & -\omega \\ \omega & \mu \end{array}\right)\left(\begin{array}{c} x\\ y \end{array}\right)$$

where *x* and *y* are the most slowly decaying eigenvectors of the system’s state variables, transformed so that the steady state lies at the origin. When *μ* < 0, the solutions to this equation spiral into the origin, completing one revolution in a time 2*π*/*ω*, and losing half of the radial amplitude in a time −ln 2/*μ*.

To simulate the behavior of this system in a fluctuating environment, we can add a driving term representing an external signal with frequency Ω and study the behavior of the resulting equations:

$$\frac{d}{dt}\left(\begin{array}{c} x\\ y \end{array}\right)=\left(\begin{array}{cc} \mu & -\omega \\ \omega & \mu \end{array}\right)\left(\begin{array}{c} x\\ y \end{array}\right)+I\left(\begin{array}{c} \cos\;\Omega t\\ \sin\;\Omega t \end{array}\right)$$

The linearity of this equation allows an exact solution. At long times, the system will show sinusoidal oscillations at the same frequency as the driving term. Assuming *μ* < 0, the amplitude *A* of these oscillations, which is a measure of signal amplification, depends on the mismatch between the natural frequency *ω* and the driving frequency Ω as:

$$A=\frac{I}{\sqrt{\mu^{2}+(\Omega -\omega {)}^{2}}}$$

The phase *θ* of oscillations relative to the driving signal is given by:

$$\tan\;\theta =\frac{\Omega -\omega }{\mu }$$

This analysis may be familiar from introductory mechanics or engineering. My hope here is to provide an application to biology where the perspective can be quite different: rather than trying to design a system with desired properties, we are often in the position of trying to study systems created by nature that evolved subject to unknown selective pressures. Simple mathematical arguments may be helpful in allowing us to see otherwise mysterious relationships between system properties and perhaps in inferring something about the statistics of the environment under which an organism evolved.

Some implications of the above equations are that near a spiral node, a system will synchronize with a driving signal regardless of its frequency. In this linear system, the phase, analogous to the angle of entrainment, is independent of the strength *I* of the driving signal. The selective amplification of frequencies allows the spiral node to act as a signal processing element, suppressing input signals with frequencies far from the natural frequency *ω* (figure 1(C)). The quality of this rejection of off-resonance frequencies improves as *μ* → 0. Note that although the response of the system is a symmetric function of frequency, many biology experiments are reported in terms of the oscillator period, and the signal amplification peak is an asymmetric function of period.

When *μ* is small, a spiral node system can be very effective at removing noise from the input signal (figure 1(D)). The width of the resonant peak becomes narrower, with a full width at half maximum 2√3μ. To be effective, the frequency of the input should fall into the resonant peak. But in a biological system, the time scale *ω* of the response will in general depend on conditions, such as growth rate, temperature, nutritional status, gene expression fluctuations, etc. Thus, for a given biochemical circuit there will be some finite uncertainty Δ*ω* associated with the response of the system. This argument leads to the following heuristic:

$$|\frac{\Delta \omega }{\omega }|∼|\frac{\mu }{\omega }|$$

Which indicates that when the natural frequency of a biochemical circuit is unreliable, the optimal choice of *μ* will be pushed away from 0 toward finite values. Conversely, this argument predicts that when rhythms are observed to rapidly die out in constant conditions (large *μ*/*ω*) the frequency of the biological oscillator may be expected to show increased variability and depend of external conditions such as temperature or growth rate (figure 1(E)). One illustrative example is the case of conditional rhythms at low temperatures in the cyanobacteria *S. elongatus*. This microbe shows robust ~24 h rhythms near 30 °C. As temperature drops below 20 °C, temperature compensating mechanisms break down and the oscillator period approaches 30 h. Consistent with the analysis here, these rhythms also lose stability as the system moves further off resonance with the environment. Remarkably, this loss of stability is part of a programmed regulatory process, since self-sustaining rhythms can be restored at low temperatures by altering the codon usage of the *kai* genes [10].

Because of the linearity of the spiral node system, inputs at multiple frequencies drive the system independently. Thus it is straightforward to analyze situations where undesirable noise has a non-uniform frequency spectrum (i.e. is not white noise). For simplicity, imagine that noise is concentrated near a single frequency Ω_noise_. In this case, what is the best-performing spiral node system? In general, it is no longer optimal to match *ω* to the signal frequency Ω. This is because, although shifting *ω* reduces the amplification of the signal, this is offset by a stronger suppression of the noise. To obtain an analytical expression for the effect of noise on the optimal oscillator frequency, one can write the shifted frequency as *ω* = Ω + *ε*, where *ε* should be chosen to maximize the ratio of signal amplification to noise amplification. Proceeding by using the formula for amplification above, we can find a maximum for the ratio of amplifications by differentiating with respect to *ε* and setting the resulting expression to zero. At leading order, the shift is:

$$\omega \approx \Omega +\frac{\mu^{2}}{\Omega -\Omega_{\text{noise}}}$$

Thus, high frequency noise results in an optimal spiral node that oscillates slower than the signal, and low frequency noise leads to optimal oscillations that are faster than the signal. This may have relevance for classical patterns observed in the study of circadian rhythms where the free-running periods of diurnal mammals, birds, and plants tend to be longer than 24 h, and nocturnal mammals and arthropods tends to have periods shorter than 24 h [1].

In general, the best performing spiral node systems are those where the time constant *ω* in the underlying biochemical mechanism can be made to be robustly independent of external conditions. This allows *μ* to become small, reducing the stability of the steady state, and reaping the benefit of a strongly peaked signal amplification curve. But there is an inherent contradiction in this argument! Letting *μ* → 0 implies that the amplitude of the system increases without bound, but the original argument was that the spiral node normal form would be a good description of the system sufficiently close to steady state. Furthermore, chemical concentrations cannot become negative, so additional terms must become important as the system moves increasingly far away from the steady state.

In general, higher order terms will limit the response of the system to resonant driving as *μ* → 0, preventing an infinite amplitude. Monti *et al* have studied the ability of such a system to extract a signal from noise. They conclude that, when higher order terms become important, enhanced performance is achieved by pushing *μ* to positive values through a Hopf bifurcation, creating a self-sustaining oscillation [5]. One possible explanation for the appearance of limit cycle oscillators (e.g. circadian rhythms) could be that they are actually easier to evolve than a highly underdamped spiral node in a biochemical circuit because the latter would require tuning all higher-order terms to nearly vanish.

Once a limit cycle oscillation emerges, the result is a biological oscillator whose response to weak driving is quite different from the linear model. In this situation, the amplitude of oscillations is nearly independent of the drive, instead being set by the size of the limit cycle itself. In this way, a limit cycle oscillator can serve to remove fluctuations in the strength of the input. The driving force acts now as an synchronizing cue, with entrainment or phase-locking occurring when the driving frequency is sufficiently close to the natural frequency. The entrained phase of the oscillator will in general depend on the drive strength, again unlike a linear oscillator [8]. These properties which allow amplitude normalization and tunable entrained phase may provide additional benefits to living organisms, favoring their evolution [7].

### 2.4. Concluding remarks

To summarize, a simple dynamical systems analysis suggests that there may be unappreciated patterns in biological rhythms that may be tested in future experiments. Uncertainty and unreliability in biochemical mechanisms tend to favor dampened rhythms. These perform modestly in separating signal from input noise but are robust in the sense that their performance is not compromised by either variability in parameters or internal noise [6]. When biochemical mechanisms are precise, performance can be enhanced by relieving the damping on the system, ultimately exposing nonlinearities in the system and creating self-sustained oscillations. Simple arguments suggest that the optimal choices of parameters will depend on the spectrum of noise in the environment, in general pushing the system slightly off resonance to better suppress noise.

Many unanswered questions remain. Does the ‘just-so’ story above describe the actual evolutionary trajectory of biological rhythms? What is the optimal design of a limit cycle oscillator in the presence of a given noise spectrum? Perhaps most importantly, are damped rhythms widespread in nature that may have received less attention because their properties can tolerate more variability?

### Acknowledgments

MJR was supported by NIH R01 GM135382 and a Howard Hughes Medical Institute Faculty Scholar award.

## 3. Energetics of rhythmic feeding

### 3.1. Status

Daily circadian rhythms of feeding/fasting and wakefulness/sleep are coordinated by the light-responsive pacemaker cells in the suprachiasmatic nuclei (SCN). SCN neurons communicate through secreted factors and projections onto nearby regions to synchronize clocks in peripheral organs with the light–dark cycle. A major output of the SCN are hypothalamic hunger and energy sensing neurons, suggesting that these regions participate in the entrainment of peripheral tissue clocks. Tissue-specific clocks can also be entrained by a variety of hormonal, temperature, and nutrient signals, such as timing of feeding. Genes controlled directly by the molecular clock as well as through clock interactions with tissue-specific transcription factors regulate metabolic rhythms of respiration, ATP production, and metabolic pathways. In turn, the rhythmic function of clock and collaborating transcription factors lead to oscillation of gene expression, translation, and protein processing, which induce alternation between anabolic and catabolic processes across tissues. Importantly, energy status and metabolites also feedback to the circadian clock to fine tune metabolic programming in cells. These bidirectional interactions between the circadian clock and metabolism are critical to coordinate energy balance throughout the day, and disruption of this crosstalk underlies metabolic disease.

Genetic evidence indicates that disruption of the molecular circadian clock is strongly linked to the development of metabolic diseases. *Clock* mutant mice fed high fat diet (HFD) display altered feeding rhythms accompanied by hyperphagia and metabolic syndrome. Similarly, *Bmal1* mutant mice have impaired glucose homeostasis. These observations highlight the significance of an intact molecular clock in regulating metabolic rhythms and whole-body energy homeostasis. Disruption of the clock through housing mice under constant light conditions also leads to glucose intolerance and elevated adiposity. Chronic low-grade inflammation of adipose tissue driven by NF-*κ*B is a hallmark of metabolic syndrome during obesity. Interestingly, mice exposed to light at night also have an exaggerated inflammatory response to the pro-inflammatory stimulus lipopolysaccharide. Recent data indicates the p65 subunit of NF-*κ*B represses transcription of CLOCK/BMAL1 target genes through binding to the promoters of genes encoding clock repressors in the liver [11]. HFD feeding leads to reduced clock gene expression in a tissue-specific manner, particularly in visceral adipose tissue. However, it is unknown whether interactions between NF-*κ*B and the circadian clock in visceral adipose tissue drive chronic inflammation and insulin resistance.

Timing of feeding and disruption of the sleep/wake cycle are key determinants of metabolic health. In nocturnal animals such as mice, much of the excess caloric intake during *ad libitum* HFD feeding occurs during the light period. Restricting feeding to the light (inactive) period results in weight gain and the development of metabolic syndrome [12]. However, restricting feeding to the dark (active) period protects mice from hepatic steatosis, glucose intolerance, and weight gain, compared to isocaloric feeding during the light. This indicates time restricted feeding (TRF) is beneficial, independent of caloric consumption. In humans, mistimed feeding as occurs during shift work, jet lag, and sleep disorders may lead to circadian desynchrony through resetting peripheral tissue clocks. Mistimed feeding could disrupt multi-organ metabolic rhythmic programming in anticipation of normal feeding times and lead to weight gain and metabolic disease. However, the molecular mechanisms underlying the metabolic benefits of TRF remain unclear.

### 3.2. Current and future challenges

Understanding the link between the circadian clock, metabolism, and feeding time are critical to developing therapies that utilize TRF to promote metabolic health and reduce obesity. Going forward, it will be critical to determine which cell types and metabolic pathways contribute to the improved metabolic health during TRF. The beneficial effects in response to eating during the optimal time likely involve coordination between multiple tissues including the pancreas, liver, adipose, intestine, and muscle. In some peripheral tissues, such as the liver, rhythmic gene expression is programmed in response to feeding time, whereas other tissues are primarily entrained by light. This demonstrates the complexity of the system-wide response to time-restricted feeding.

Much of the focus in time-restricted feeding has been on the liver, while less is known about rhythmic changes in response to feeding time in other peripheral organs important in energy homeostasis. However, it was recently demonstrated that *Bmal1* and *Reverbα/β* in the liver are not required for the reduced weight loss, decreased adiposity, and restored glucose homeostasis driven by restricting feeding to the dark period as compared to *ad lib* feeding [13]. This indicates other tissues may be responsible for the beneficial effects of TRF. Global metabolite profiling comparing chow-fed and HFD-fed mice revealed heterogeneity in metabolites across tissues with a loss of lipid oscillation in BAT after HFD feeding [14]. This data along with the prominent change in adiposity during TRF suggests adipose tissue metabolism may play a role in mediating metabolic health during rhythmic feeding. Indeed, restricting HFD to the dark period induces *Ucp1* expression in BAT and reduces white adipocyte hypertrophy. BAT thermogenesis is regulated in a circadian manner and is highest during the active period [15]. Restricting feeding to the light period leads to reduced body temperature during the dark period, suggesting reduced thermogenesis [16]. This suggests adipose tissue thermogenesis and lipid metabolism may underlie metabolic benefits driven by eating at the optimal circadian time of day.

Another major challenge in translating metabolism research from the rodent to humans is that research is typically performed during the light period, during nocturnal rodents’ sleep phase. Future work on the interplay between energy balance and diet should focus on metabolic mechanisms in a time-of-day dependent manner.

### 3.3. Advances in science and technology to meet challenges

Two recent studies in humans indicated TRF improves glucose homeostasis and blood pressure in pre-diabetic men independent of weight loss [17, 18]. However, the mechanisms underlying how synchronizing feeding time with circadian rhythms benefit metabolic health remain poorly understood. The recent development of automated feeding equipment that controls for amount, duration, and timing of food availability greatly advances the ability to study the interplay between circadian rhythms and timing of feeding in mice [19]. Cistromic profiling in different tissues revealed that clock components bind to distinct tissue-specific enhancer sites, highlighting the importance of studying circadian clocks in a tissue- and cell-specific manner. CLOCK/BMAL1 co-localize with the pancreatic transcription factor PDX1 in beta cells, distinct from the liver-defined binding sites that program metabolic networks [20]. The use of inducible genetic CRE models that target individual cell populations combined with floxed alleles provides temporal and spatial ablation of genes, which is advantageous over constitutive whole-body gene knockouts. Future work using tissue-specific inducible genetic deletion is critical to elucidate the heterogeneity of cellular responses during TRF.

### 3.4. Concluding remarks

Oscillations in oxidative and reductive metabolism are synchronized by circadian clocks in anticipation of light/dark and feeding/fasting cycles. During feeding, metabolic pathways are coordinated across multiple tissues in order to achieve organismal homeostasis. Elucidating circadian clock function in a tissue-specific manner is essential to understanding how circadian desynchrony of feeding time participates in metabolic disorders.

### Acknowledgments

We thank Dr Jonathan Cedernaes and all members of the Bass lab for helpful discussions. This research was supported by the National Institute of Diabetes and Digestive and Kidney Diseases (NIDDK) grants R01DK090625, R01DK113011, R01DK100814 and R01DK050203, The National Institute on Aging grant P01AG011412, the Chicago Biomedical Consortium S-007 and the University of Chicago Diabetes Research and Training Center grant P60DK020595 (JB).

### Data availability statement

Nonewdata were created or analysed in this study.

### Conflict of interest

The authors declare that they have no competing financial interests.

## 4. Extracellular signals and dynamic intracellular change

### 4.1. Status

Cells have been selected for change. Even microbes use current signals to prepare for the future [21], and in our own cells circadian rhythms have hard-coded such preparation into a daily occurrence. In natural environments, be that a human tissue or as part of a microbiome, extracellular signals are likely to be multifarious, simultaneous, and continually varying. Yet it is only recently that microfluidic technology has allowed us to overcome the technical challenge of mimicking such signals.

**Figure 2.**
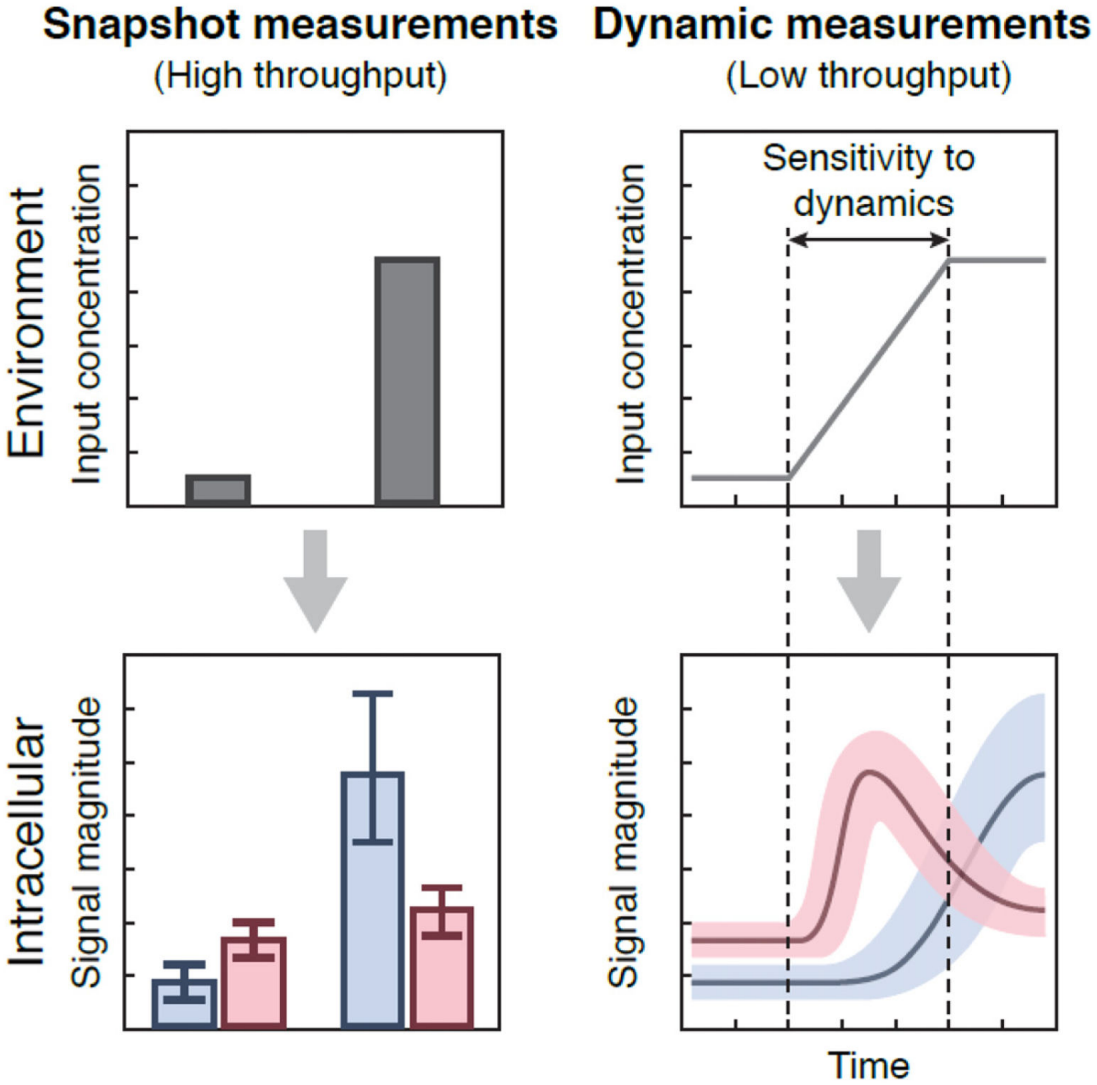
Some cellular responses are only revealed by dynamic inputs. In steady-state experiments, the blue but not the red molecule appears to respond to the input. A dynamic input, however, shows that the red molecule responds as strongly as the blue molecule, but to the input’s time-derivative not its absolute value.

Signaling networks should perform best in natural environments, and using dynamic inputs is proving a powerful means to understand their internal logic [22] (figure 2). There are mutants in the signaling pathway responding to hyperosmotic stress in budding yeast that only become distinguishable from wild-type when exposed to time-varying inputs [23], and some stress responses in bacteria respond not only to stress but also to its rate of increase [24]. Higher organisms may even regulate extracellular environments to become dynamic and use oscillatory levels of cytokines to selectively entrain signaling pathways [25].

Intracellular responses are dynamic too, and only a step change in an extracellular concentration can generate complex intracellular behavior. The levels of second messengers, such as calcium and cAMP, can spike or oscillate; metabolic cycles might change phase; and some transcription factors pulse in and out of the nucleus.

We are only beginning to understand why cells might use such dynamic signaling over steady-state responses. Dynamic responses are potentially quicker than waiting for steady-state behavior and also may carry more information because not only the amplitude but also the timing of the response can be used [26, 27]. Signaling pathways at steady-state appear to encode only enough information to distinguish between two types of environment, but the information substantially increases if the downstream biochemistry can sense the response’s dynamics. Encoding different extracellular signals in the dynamics of signaling molecules can also coordinate downstream responses. A transcription factor that pulses in and out of the nucleus with a frequency but not amplitude that changes in different environments will always have the same concentration when in the nucleus, causing all regulated genes to respond together [28].

### 4.2. Current and future challenges

Characterising dynamic behavior requires finding suitable reporters. Their quality constrains the time resolution, the numbers of cells monitored, and the numbers of variables measured. Reporters must respond on appropriate time scales to capture dynamics, be sensitive to short acquisition times, and sufficiently responsive to excitation to limit photo-toxicity. Although monitoring transcription using RNA-binding proteins and signal transduction through nuclear translocation are both fast, each can potentially perturb intracellular dynamics.

A second challenge is choosing the input. Typically, we do not know the natural signals under which cells have evolved, if the input should change with time, or if it should appear alone or co-vary with others. A dynamic input greatly increases the number of variables—up to one for each time point. Exploring such a vast space is daunting, and without efficient methods we must make do with low sampling.

Studying individual cells itself raises problems because cellular context can determine behavior. As well as the inherent stochasticity of biochemistry, cellular history—how cells were prepared and previous exposure to signals—and cell state, such as phases of the cell cycle, metabolic cycle, or circadian cycle, can alter responses and confound interpretation. To make matters worse, we often do not have reporters for such endogenous rhythms. This variation means that we need quantitative methods to compare collections of time series. For example, there is no standard procedure to determine statistically significant differences between two sets of time series, such as for a wild-type and mutant.

Although microfluidic technology has become indispensable, the device’s design could bias intracellular dynamics. Often a device favors particular cellular shapes, and being confined can stress cells and alter gene expression. As the experiment runs, the cells under study can become unrepresentative of natural populations. For example, multiple devices trap cells but allow offspring to escape, and imaging for say eight generations means that the trapped cells constitute only 2^−8^ of a growing population. Further, polydimethylsiloxane (PDMS), the polymer often used in devices, can influence cellular behavior and absorbs hydrophobic molecules, potentially distorting inputs.

### 4.3. Advances in science and technology to meet challenges

Better reporters of intracellular activity would be transformative. Cross-talk between fluorophores limits most studies to two reporters, giving only a blinkered view of the response. Although we can control some signaling, such as kinases made sensitive to 1-NM-PP1 and through targeted degradation and optogenetics [29], we cannot measure *in vivo* the drivers of cellular decision-making—active kinases and phosphatases. Non-perturbative methods to follow RNAs and cellular cycles as well as reporters to quantify cellular context—levels of cofactors like NAD^+^, of second messengers, and of energy (the ATP to ADP ratio, proton motive force, and membrane potential)—are all essential.

To mimic natural environments, we need reproducible control of the dynamics of inputs, the ability to apply multiple inputs, both simultaneously and sequentially, and optimization to efficiently explore the space of inputs. Chemical methods to reduce the hydrophobicity of PDMS, like silanization, will both prevent microfluidic devices perturbing inputs and enable new dyes as intracellular reporters.

Progress is needed on two bottlenecks: extracting information from time-lapse experiments and efficient means to search and share time-lapse data. Many laboratories develop in-house software for phenotyping cells that is too customized for data from elsewhere, and results must often be manually corrected. Advances in convolutional neural networks should fix both problems. With sufficient training data, these algorithms work better and faster than traditional approaches, and techniques for transfer learning are facilitating sharing [30]. Agreeing on a standard format for storing images, annotations and associated meta-data will allow both exchanges and the meta-analyses needed for ‘whole-cell’ modeling.

Perhaps the most impact will be from combining time-series experiments with single-cell’omics. If a group of cells that has displayed a particular dynamic phenotype could be selectively extracted from a microfluidic device, then single-cell transcriptomics and proteomics will give numbers of reporters impossible to achieve with fluorescence, albeit at one time point. We will then be able to determine how the dynamics of inputs, movements of transcription factors, individual cell physiologies, and phases of endogenous rhythms in the recent past affect current programmes of gene expression.

### Acknowledgments

We acknowledge funding from the BBSRC and the Leverhulme Trust.

## 5. The role of timing in biological perception and actuation

### 5.1. Status

Physicists often study biological networks as closed systems that evolve according to their own autonomous nonlinear dynamics. For example, a cell observed under the microscope will reliably move through stages of growth, DNA replication, and mitosis, taking an observer through each step of the cell cycle. Such a system lends itself well to modeling, and various emergent properties can be predicted: the stable states the cell finds itself in for extended periods of time, the speed at which it moves from state to state, and the period of the cycle.

But our picture of the cell cycle as a closed system is incomplete, as cell growth and division are highly responsive to environmental cues: local cell density, nutrient availability, the presence of permissive growth factors, and even subtle variations in temperature that elicit a biological stress response can dramatically alter or halt cell cycle progression.

It may thus be more fruitful to view the cell not as a closed, autonomous system but as a set of signal processing devices. We may borrow concepts from information theory, circuit design, and control theory to ask what dynamic filters, relays, and data compressors may underlie the cell’s response to environmental cues [31]. How are useful and pertinent signals deciphered from a sea of external chemical and mechanical cues? Is there a ‘code book’ for intracellular signal transmission?

### 5.2. Current and future challenges

**Figure 3.**
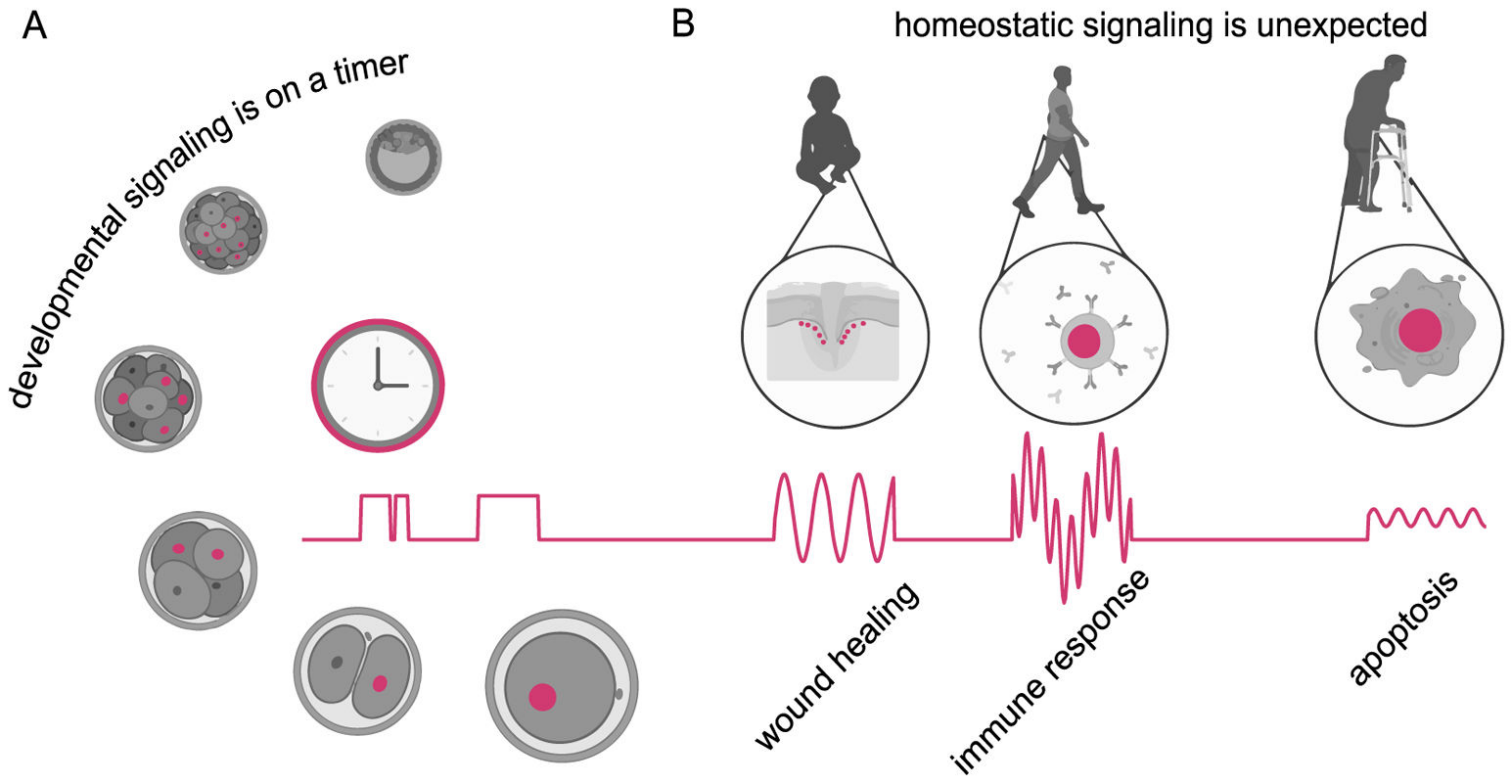
Developmental and homeostatic signaling. Throughout development (A), signaling pathways (activity shown in purple) can potentiate cell fate decisions, and do so in a very deterministic and stereotyped manner. However, during homeostasis (B) and throughout the organism’s life, events such as wounding, immune responses, and programmed apoptosis in response to stressors can activate the same pathway in a nondeterministic fashion.

A few key biological processes are emerging as ideal context for studying cellular signal processing (figure 3). One is embryo development, where almost every transition is closely linked to a biological clock or timer. After all, cells have a limited time to migrate, divide or differentiate before the embryo proceeds to its next developmental stage.

For example, the *Drosophila* embryo’s first fourteen nuclear cycles occur under extremely stereotyped time intervals and after approximately 3 h culminate in the profound cell movements associated with gastrulation. The formation of the segmented body plan and three germ layers must be completed on this timeline, requiring fast (minutes–hours) signaling events and transcriptional responses. Supporting this view, we found that Erk-dependent differentiation into gut endoderm and neural ectoderm was limited to a critical time window between nuclear cycle 10 (when nuclei move to the embryo’s surface and can receive Erk-dependent signaling) and gastrulation [32]. The total duration of Erk signaling delivered in this narrow, 90 min time window proved to be essential for cell fate specification.

A second key context for dynamic signaling can be found in the maintenance, homeostasis, and repair of adult tissues. Numerous signaling pathways that were crucial to embryo development are again repurposed in the adult organism, where the objective is not the timely progression through embryogenesis but rather continuous tissue- and organism-level homeostasis.

Yet despite a high degree of molecular conservation, the requirements for homeostatic signaling are quite different than those in development. Homeostatic signaling must be sensitive, detecting a single defective cell among millions of normal ones; in contrast, inductive developmental cues can be produced at high concentrations. Homeostatic pathways must also respond to inputs with a huge range of unpredictable spatial distributions and timescales (e.g., wounds can be tiny or huge, acute or chronic), whereas developmental cues usually occur in predictable time windows. A sophisticated degree of information processing is essential to meet these varied constraints.

Interestingly, mounting evidence suggests that cell signaling in adult tissues also possesses its own complex spatiotemporal behavior [33]. This may include pulses of pathway activity, traveling waves across a tissue field, or switch-like and irreversible cell–fate transitions (e.g., apoptosis). In some cases, it is not obvious which stimuli are responsible for the observed the signaling dynamics, such as in the case of the tumor suppressor p53, which exhibits dynamic activity even in the absence of any externally-applied stress [34]. It remains an open question in the field to determine which variations in signaling are due to actual changes in an upstream input as opposed to autonomous, stochastic activation of the pathway.

### 5.3. Advances in science and technology to meet challenges

A first key to cracking the signaling code lies in systematically varying external signaling cues and monitoring the cell’s resulting intracellular states. This approach has been taken with regards to neural processing: Hodgkin’s and Huxley’s seminal studies on single neurons adopted an ‘input–output’ approach, plugging in transmembrane voltage as an input and measuring the resulting current flow to usher in the era of quantitative neurobiology.

However, neurobiology is far from the only context in which time-varying signaling takes place. One difficulty in porting the neuroscientist’s toolbox to cell biology has involved designing methods to accurately define and vary an external cue. Membrane potentials can be easily applied and removed with high temporal accuracy, but receptor–ligand and protein–protein interactions have proven to be more challenging to control. The recent development of microfluidics, optogenetics, and live-cell fluorescent biosensors provide a rich and growing toolbox for overcoming this difficulty. Indeed, these new tools have been applied with some success to study Rho GTPase signaling in single cells [35], to control and visualize the outcomes of NF-kB signaling [36], and to map signal transmission through the Ras/Erk signaling pathway [29].

**Figure 4.**
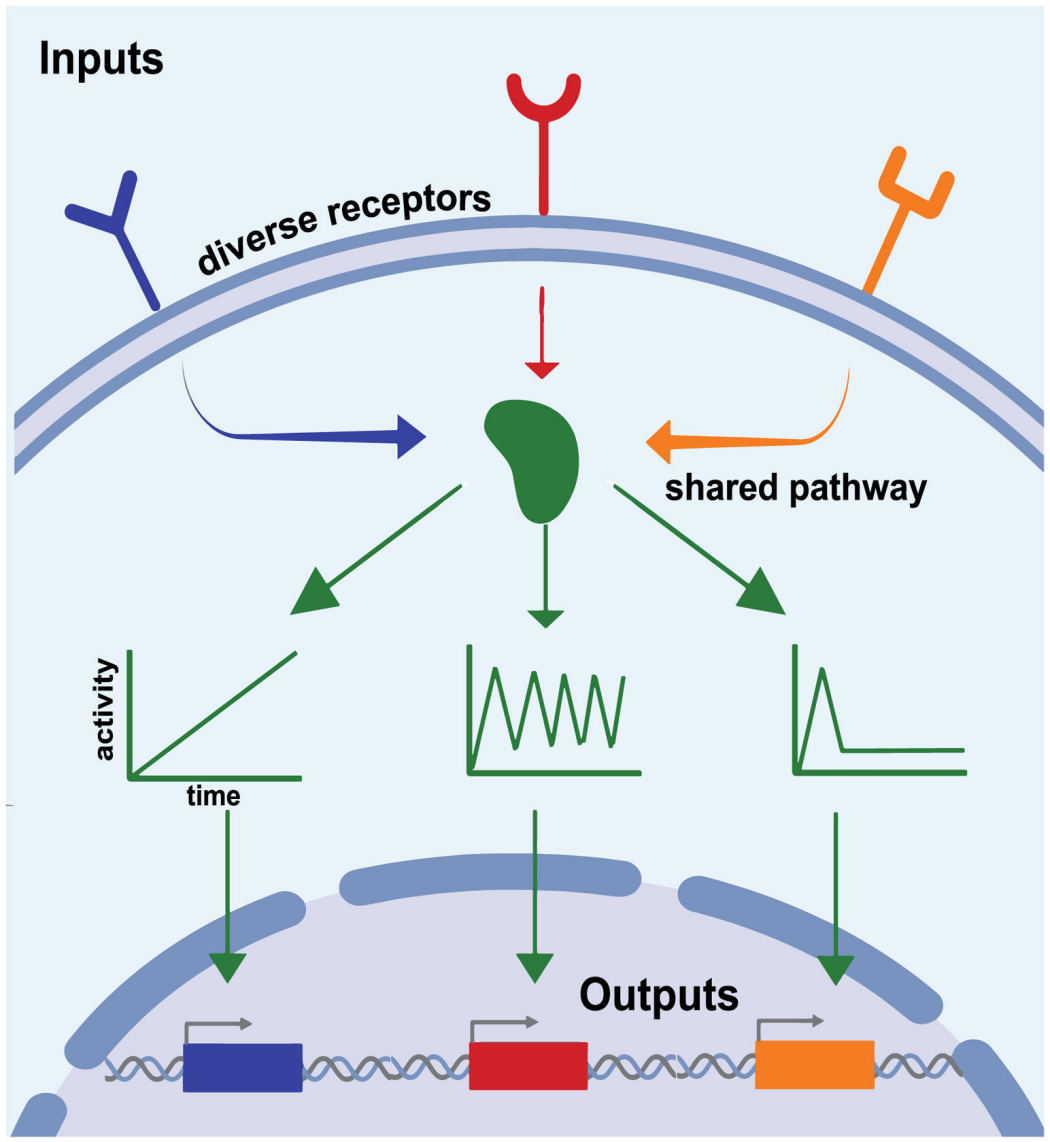
Decoding signals into appropriate genetic outcomes. Environmental signals sensed by receptors (in blue, red, and yellow) can feed into the same pathway and cause distinct gene expression outcomes (blue, red, yellow genes). One hypothesis for how this occurs is that the conserved pathway (green) can be repurposed in its dynamics to deliver constant linear activation, oscillations of activation and inactivation, or a single transient pulse of activation, all of which are read out in distinct ways.

What functional role might signaling dynamics play—is there significance to whether a pathway exhibits a sharp off-to-on transition versus periodic pulses or waves of activity? In some cases, the role of dynamics can be easily intuited. For example, an ultrasensitive off-to-on protein switch enables the cell to trigger a long-term, all-or-none change to a transient stimulus, whereas a signaling pulse arises naturally from sensory systems that adapt and desensitize to a constant stimulus. However, other dynamics observed in signaling pathways are not so easy to explain. For many intracellular signals (e.g., p53, Crz1, Erk, Ascl1, and Msn2), different pulsatile dynamics emerge from different inputs, suggesting that they may represent a coding strategy to share a single protein circuit between multiple cellular response programs [28, 37, 38]. By directly controlling pathway dynamics and measuring responses, it may be possible to deconstruct these coding strategies (figure 4).

A case study for understanding signal multiplexing is the metazoan Ras/Erk pathway. This pathway has been found to respond to a wide range of inputs, including a range of growth factors, cell–cell contact, and even mechanical force. These inputs activate a phosphorylation cascade that culminates in the activation of Erk and its translocation into the nucleus, where it potentiates a cellular response through gene expression. Using an optogenetic approach that relies on light-controlled protein heterodimerization, Toettcher *et al* were able to precisely control activation of the signaling pathway, demonstrating that it acted as a low-pass filter that responded more readily to longer input durations [29]. In subsequent work, Wilson *et al* studied the resulting expression of Erk-stimulated immediate-early genes (IEGs) [39]. Although the requirement for Ras/Erk signaling to drive IEG expression was demonstrated decades earlier, using optogenetics to deliver time-varying signals led to the discovery that genes act as band-pass filters for Erk signaling, suggesting that two separate filter layers operate between Ras activation and the eventual accumulation of target gene products.

Accumulating evidence demonstrates that Erk signaling dynamics have real consequences in disease and development. It had long been known that aberrant growth factor signaling led to uncontrolled growth and cancerous phenotypes. However, it has been broadly assumed that uncontrolled growth is the result of constitutive, high signaling activity independent of any time-varying stimulus. Surprisingly, it was recently observed that dynamics play a role in mutation-induced signaling as well. Using live cell reporters and optogenetics, Bugaj *et al* quantified signal transmission in tumor cells harboring a BRaf P-loop mutation (G469A) [40]. The authors demonstrated that mutant BRaf extended Erk signaling long after stimuli had bene removed, leading to chronic misinterpretation of dynamic stimuli and expanding the set of stimuli capable of driving cell proliferation. Recently, we also found that Ras/Erk dynamics also play a crucial role in *Drosophila* embryo development. Using optogenetic control over Ras, we found that simply increasing the duration of pathway activity could specify a single cell population to adopt fates from two different germ layers (gut endoderm vs neural ectoderm) [40].

Although progress has been made to define the code book of Ras/Erk dynamics and their resulting cell–fate outcomes, much remains unknown. What is the molecular origin of the seemingly spontaneous, excitable Erk pulses that arise in so many cellular contexts? Which molecular circuits interpret and filter dynamics into different gene expression programs? Both the encoding machinery that initiates dynamics and the downstream decoding machinery remain undefined.

### 5.4. Concluding remarks

A grand unifying theory of dynamic encoding should be able to explain how dynamics can be interpreted on a molecular level, and the situations in which they offer benefits that alternative strategies cannot provide. There are other promising candidate encoding–decoding strategies: combinatorial logic, where responses are defined by two or more inputs; or the action of morphogens, where a substance’s concentration is interpreted into a defined cellular response. One attractive hypothesis is that encoding information in the timescale of protein activity (rather than protein concentration) enables more accurate decision-making, because biochemical timescales (e.g., dissociation, degradation, or diffusion rates) are less heterogeneous between cells and over time than are protein levels. Circuits that measure a process’ timescale may thus perform more precise, reproducible computation.

A second possibility is that by coupling excitable, noisy dynamics between many individual cells, a tissue may be able to sense weak inputs that no single cell could accurately detect on its own. This phenomenon, related to the physics concept of stochastic resonance, has been predicted to enable robust input sensing in populations of neurons and oscillations of cortical actin polymerization [41, 42]. Erk dynamics, too, may entrain to sub-threshold EGF doses [43].

The source of dynamics is also a mystery: how do complex signaling dynamics emerge from constant environmental input? It is unlikely that the same sets of proteins give rise to dynamic responses across diverse signaling pathways and contexts, yet a small number of network architectures may recur across these contexts. If so, are these networks moulded, trimmed, and optimized over evolutionary time to shift their dynamic responses based on the relevant stimulus strengths and biological timescales in each case? Using the quantitative tools and systems-level approaches described here, the construction of a ‘periodic table’ of the signaling modules utilized by living systems may be possible.

### Acknowledgments

We thank Alexander Goglia for insightful discussion and comments on the manuscript. This work was also supported by NIH Grant DP2EB024247 and NSF CAREER Award 1750663 (to JET) and an NIH predoctoral fellowship F31075398-01 (to SGJ).

## 6. Eliciting potent antibodies against highly mutable pathogens by vaccination

### 6.1. Status

Effective vaccines protect humans from a particular disease-causing pathogen by eliciting potent immune responses that are specific for the pathogen, and which can be rapidly recalled upon infection. Abs are an important component of such immune responses. B cells produce Abs by a Darwinian evolutionary process called affinity maturation (AM) [44]. Most B cells have a surface receptor (BCR) that is distinct from those of other B cells. B cells whose receptors can bind sufficiently strongly to the surface proteins of a pathogen or vaccine component (called antigens) can seed structures called germinal centers (GCs) in lymph nodes. In GCs, the receptors of B cells mutate rapidly, followed by a series of steps which stochastically select for those that bind more strongly to the antigen. Some positively-selected B cells morph into Ab-secreting plasma cells and exit the GC, while the majority are recycled for further rounds of mutation and selection. Thus, as time ensues, Abs with increasing strength of binding, or specificity, for the antigen evolve that can then neutralize the pathogen.

Highly mutable pathogens (e.g., influenza, HIV) rapidly evolve their surface proteins, so Abs specific for regions of these antigens that are mutable cannot protect against diverse viral strains. The antigens contain some regions that are relatively conserved for functional reasons, but they are usually surrounded by highly variable regions, thus complicating Ab binding to the conserved regions. Recently, Abs (present in low numbers) have been isolated from some individuals infected with HIV and influenza that can neutralize diverse viral strains [45]. These broadly neutralizing antibodies (bnAbs) bind to the conserved antigenic regions. This shows that the human immune system can evolve such ‘generalists’, albeit inefficiently. Many experimental efforts have been launched to devise strategies to elicit bnAbs in diverse people upon vaccination [45]. Fundamental studies of how to teach the immune system to evolve bnAbs have also been reported [46-51]. Much more work is required to obtain a deep understanding of this complex problem at the intersection of statistical physics, immunology, and evolutionary biology. Progress can help guide the design of life-saving vaccines.

### 6.2. Current and future challenges

**Figure 5.**
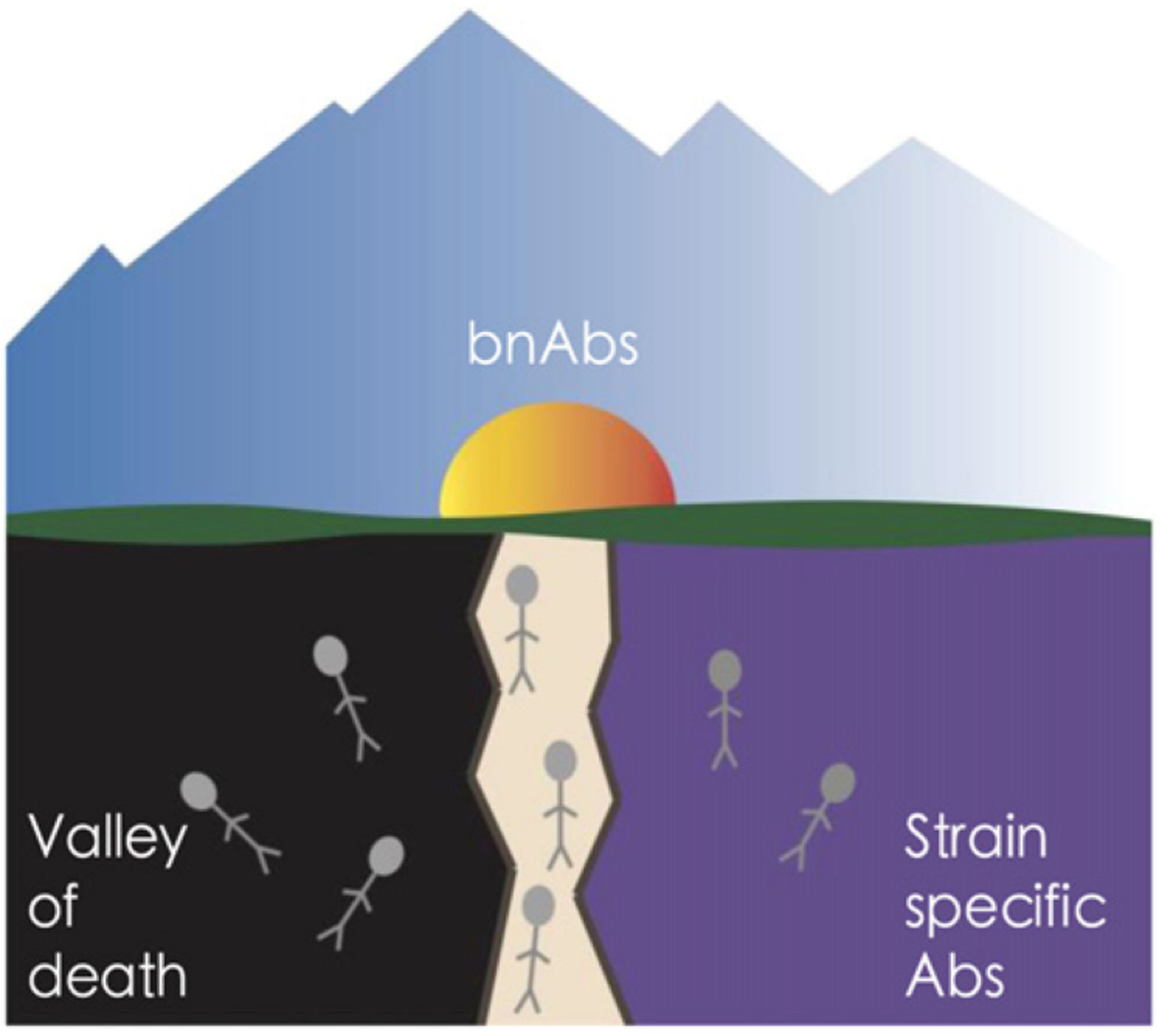
Evolution of bnAbs. Vaccination with a single antigen produces only strain-specific Abs, whereas more complex, conflicting selection forces are required to evolve bNAbs (see main text). These conflicting selection forces frustrate the normal process of AM, and can lead to B cell death in GCs. Hence, bnAb evolution walks a fine line, requiring selection forces that are optimally imposed during vaccination.

The process of AM will generate only strain-specific Abs upon vaccination with a single antigen. The selection forces that need to be imposed on AM to evolve bnAbs must therefore be comprised of multiple variant antigens that share the conserved regions, but differ in the variable regions. In the early stages of AM, it is unlikely that B cell receptors have evolved strong interactions with the conserved regions, and so binding to variant antigens sufficiently strongly to be positively selected is likely mediated by binding to both variable and conserved regions. As the antigens have different variable regions, they represent conflicting selection forces [46, 47]; features that are favorable for ‘local stability’ (binding well to one antigen) are unfavorable for ‘global stability’ (binding well to diverse antigens). This facet of the system is analogous to frustration in physical systems [52], but because AM is driven out of equilibrium, the frustration due to conflicting selection forces can result in B cell death in the GC (figure 5) [46, 47].

The presence of conflicting selection forces or frustration during AM poses a number of significant current and future challenges that need to be addressed, and requires fundamental advances in our understanding of AM. In particular, how should frustration be quantified in terms of simple metrics of fitness, and are there optimal temporal patterns of frustration that promote bnAb evolution? If so, what are the mechanistic underpinnings that define such optimal patterns in terms of the extent to which the system is driven out of equilibrium as time ensues? In addition, how do the temporally varying selection forces affect the diversity of evolving B cells, and are there optimal levels of diversity that promote trajectories that evolve bnAbs? How important is clonal interference? More broadly, are there general principles pertinent to evolutionary biology and statistical mechanics of learning that can be gleaned? How can these principles be translated into efficient vaccination protocols? Insights obtained from studies pursuing these questions must be tested using animal models, and iteration between such studies and theory/computation will provide the principles that we seek.

### 6.3. Advances in science and technology to meet challenges

Theory and computation have played a key role in helping to address some of the challenges outlined above. Studies of how evolving systems can be progressively pushed out of equilibrium to evolve bnAbs has begun to shed light on how different temporal frustration patterns influence the outcomes of AM [46-48]. Mechanistic insights from such studies can be tested in settings where evolution occurs rapidly in a real biological system, rather than in contrived laboratory systems [53].

**Figure 6.**
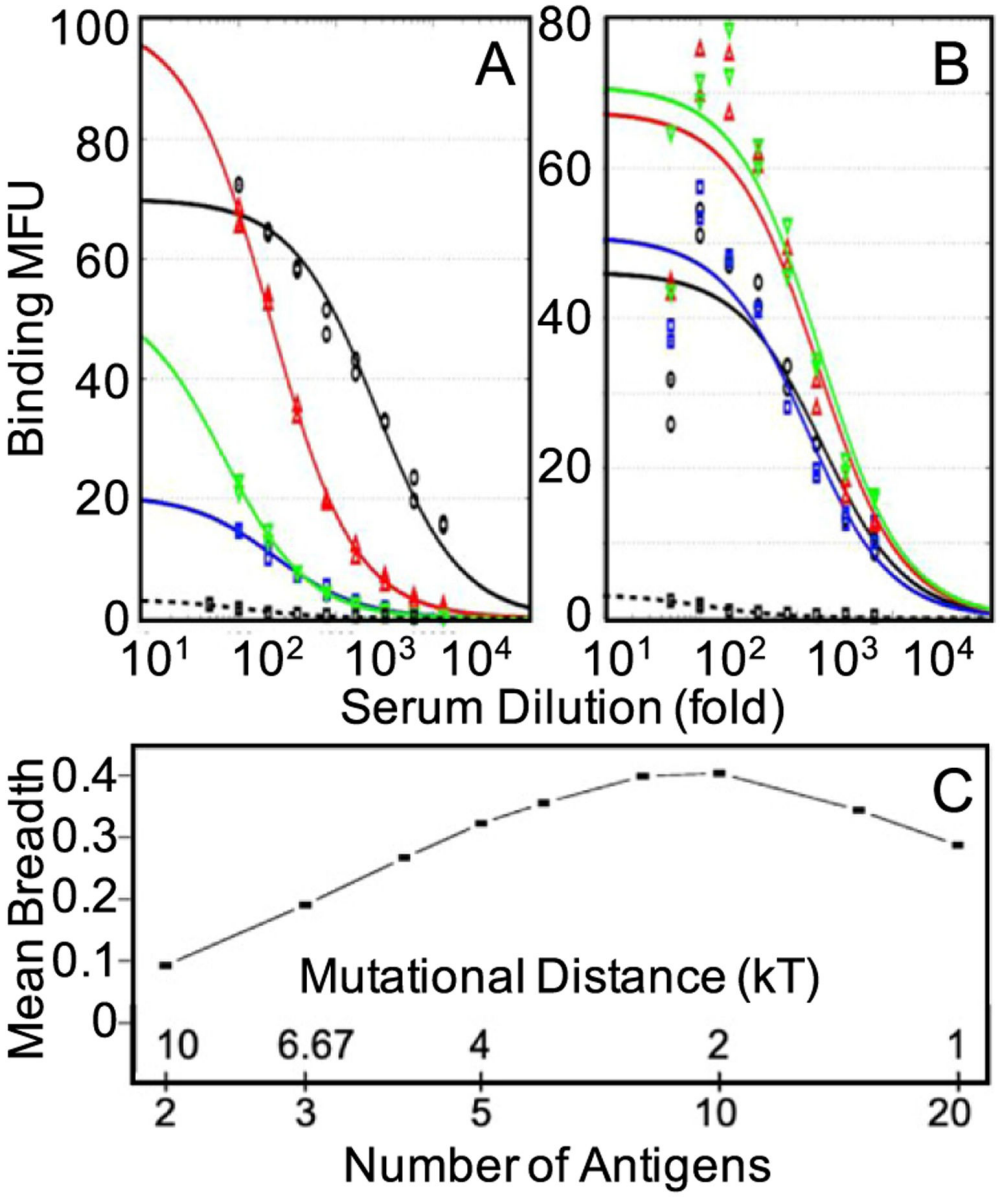
Principles gleaned from studies of AM. (A) Cocktail immunization with four antigens (solid lines; dotted lines = control), leads to high variability in binding of the produced Abs for those antigens. (B) Sequential immunization of the same antigens results in high Ab binding to all antigens. (C) Optimal frustration conditions exist for cocktail administration, including the number of antigens and mutational distance between them. Figures were adapted from references [3] (plots A, B) and [4] (plot C).

Computational studies have shown that if the variant antigens that are administered differ significantly in their sequences, and are present simultaneously in the GC, death is very likely [46]. Sequentially administering the same variant antigens, which corresponds to a temporally varying pattern of imposed selection forces, has been shown to be more effective (figures 6(A) and (B)) [46]. It has also been proposed that an optimal level of frustration exists when the variant antigens are present simultaneously in the GC (figure 6(C)) [47]. Too high a level of frustration leads to B cell death, and too low a level results in positive selection of many B cells. Thus, the GC reaction rapidly ends before B cells can acquire the necessary numbers of mutations to evolve bnAbs. A recent theoretical study reports on an interesting effect of a temporal pattern of oscillating selection forces with increasing frequencies [48]. But, our understanding is still highly incomplete, and important strides forward in the development of a theory of how Abs learn patterns in an evolving environment during AM are required.

Further progress will require many additional advances. For instance, how different should the variable regions of the variant antigens be in order to achieve a desired level of frustration, and how can the temporal pattern of frustration be manipulated to generate the optimal variation of B cell clonal diversity to evolve bnAbs? Translating the principles derived above into designs of specific variant antigens will require atomistically detailed simulations and close coupling to efforts of biologists engaged in immunogen design. Can such studies be reliably done? The first targets for translating principles to practice could be HIV and influenza.

### 6.4. Concluding remarks

Despite many remaining challenges, the real possibility of generating universal vaccines against highly mutable pathogens by eliciting bnAbs has focused a great deal of interest and research toward accomplishing this goal. Solving this fundamental problem at the crossroads of statistical physics, immunology, evolutionary biology, and learning theory, will require synergy between the efforts of statistical physicists, biophysicists, and immunologists. We anticipate that these efforts will lead to substantial advances in our understanding of evolution in time-varying environments. Such advances will have far-ranging implications for preventing diseases caused by infectious pathogens, a challenge that has plagued humanity since antiquity.

## 7. Dynamics in fluctuating and evolving environments

### 7.1. Status

The capacity to adapt to changing conditions is a hallmark of living systems. Examples of biological adaptation range from the allosteric regulation of enzymes in seconds to the fixation of genes in populations in the course of years. Despite differences of scales, mechanisms of adaptation are all fundamentally coupled: adaptations on short time scales are subject evolution on longer time scales. Mechanisms of adaptation are therefore potentially adaptations themselves, which raises the possibility of understanding them within an evolutionary framework.

**Figure 7.**
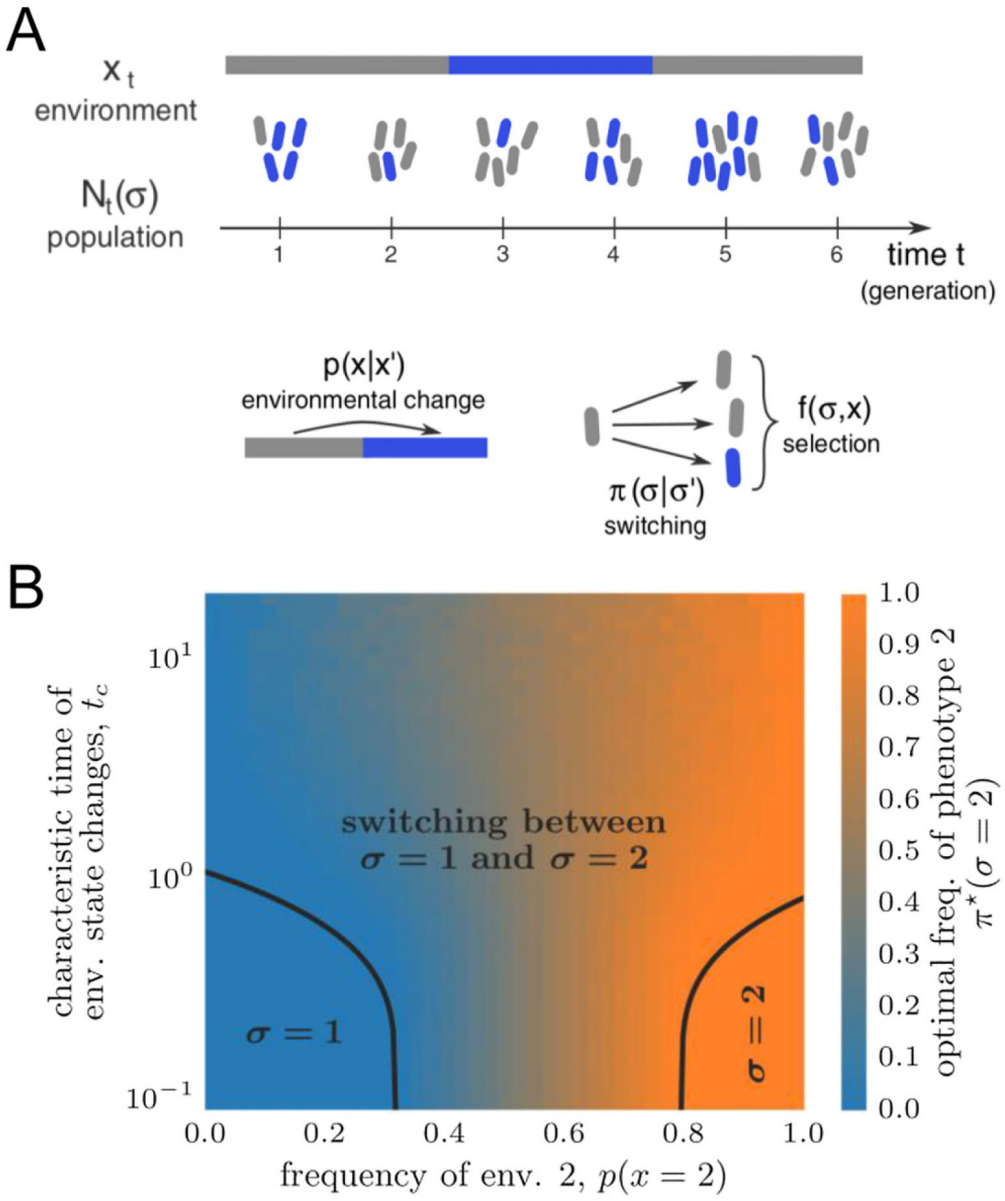
(A) Illustration of Kelly’s formalism in a simple case where the environment fluctuates between two states, representing for instance the absence (*x* = 1, in gray) or presence (*x* = 2, in blue) of a pathogen. This environment is experienced by a population of reproducing individuals that can themselves be in two states, e.g., resistant (*σ* = 1, in gray) or not (*σ* = 2, in blue). At each generation *t*, the environment has probability *p*(*x*∣*x′*) to change from state *x′* to state *x*. Each individual of the population has also a probability *π*(*σ*∣*σ′*) to change its state from *σ′* to *σ*. An individual that has switched to state *σ* then contribute an average of *f*(*σ*, *x*) individuals in state *σ* to the next generation. The dynamics is described by equations (1) and (2). (B) The strategy *π*(*σ*∣*σ′*) optimizing the long-term growth rate Λ depends on the nature of the environmental fluctuations, here the frequency of the pathogen *p*(*x* = 2) and a characteristic time of environmental change *t*_c_ that we define by *e*^−1/*t*_c_^ = 1 − *p*(1∣2) − *p*(2∣1). Taking *f*(*σ* = 1, *x* = 1) = 1, *f*(*σ* = 1, *x* = 2) = 0.3, *f*(*σ* = 2, *x* = 1) = 0.4, *f*(*σ* = 2, *x* = 2) = 1 to capture the relative costs of immunity and infection, the results show that switching between the two states *σ* = 1 and *σ* = 2 is favored only in some intermediate regime of environmental fluctuations (adapted from [58]).

One such framework, originally developed by Kelly to analyze optimal strategies in horse races [54], applies to populations of non-interacting individuals switching between pre-defined states [55]. In this framework, the individuals reproduce based on their state *σ* and on the state *xt* of the environment at time *t*, which can fluctuate independently of the population (figure 7(A)). Different strategies of adaptation amount to different probabilities *π*(*σ*∣*σ′*) of switching from an internal state *σ′* to an internal state *σ*. An optimal strategy is defined as maximizing the long-term growth rate Λ of the population

(1) $$\Lambda =\underset{t\to \infty }{\text{lim}}\;\frac{1}{t}\;\text{ln}\sum_{\sigma }N_{t}\;(\sigma )$$

where the number *Nt*(*σ*) of individuals in state *σ* at generation *t* follows

(2) $$N_{t}\;(\sigma )=\sum_{\sigma^{\prime }}\pi \;(\sigma |\sigma^{\prime })\;f\;(\sigma^{\prime },x_{t})\;N_{t-1}\;(\sigma^{\prime })$$

How optimal adaptations depend on environmental fluctuations can then be summarized in a phase diagram (figure 7(B)).

By incorporating sensing of the environment, individual noise, costs or a distinction between genotype and phenotype, this formalism rationalizes the existence of a number of puzzling biological features, including phenotypic noise, non-genetic heredity and Lamarckian mechanisms [56]. The recurrent finding is that the statistics of environmental fluctuations is a key parameter defining optimal adaptations. For instance, we developed a model of immunity where the diversity of observed immune systems is obtained by varying the frequency and duration of the occurrence of pathogens [57].

Despite important limitations, such as not accounting for population extinction, the approach formalizes the informational language that pervades informal biological descriptions and thus identifies a level of coarse-graining that reveals commonalities between systems irrespectively of specific implementations. In view of developing a theoretical understanding of living systems, extending this approach beyond its current range of application would be, in our opinion, very valuable.

### 7.2. Current and future challenges

Applications of Kelly’s formalism are currently limited by the requirements that environmental fluctuations should be independent of the population and operate on timescales much shorter than the scale over which the mechanisms of adaptation evolve. These assumptions are not applicable to many instances of biological interest.

First, empirical analyses of environmental fluctuations such as temperature or nutrient availability show both a very broad spectrum of fluctuations and long-term non-stationary trends.

Second, biological environments are shaped by the populations that experience them. On short timescales a growing population depletes nutrients from its environment while on longer timescales it may fundamentally alter its nature, a phenomenon known as niche construction. More generally, the environment of an individual comprises the other individuals from the same and different species, which are themselves evolving.

In particular, all living organisms coevolve with viruses that infect them. Viruses typically have a shorter generation time than their host and are thus able to evolve on shorter timescale than them. Hosts survive through one universal strategy, diversification, which may be either geno/phenotypic (e.g., diversity of cell receptors) or environmental (e.g., refuge in a biofilm). On long-time scales, these mechanisms to generate diversity are subject to evolution as are the mechanisms through which viruses adapt. Should we expect a convergence or a separation of time scales between host and viral adaptive strategies? What is the interplay with intrinsic time scales such as generation time and extrinsic time scales such as seasonal changes? Can we justify our previous model [58] where the dynamics of pathogens is independent of that of the host population?

**Figure 8.**
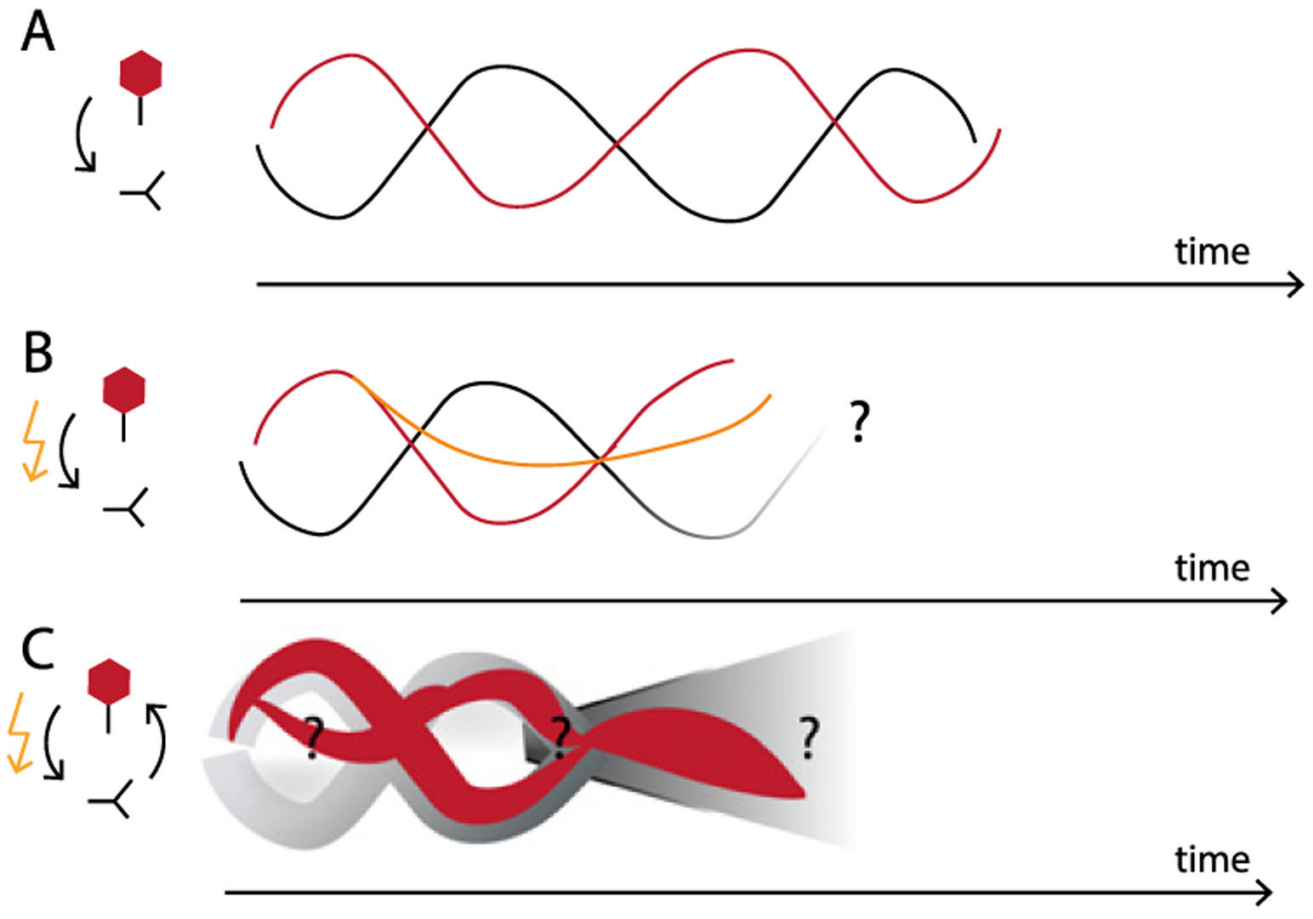
Scenarios of co-evolution between host and viral populations. (A) Kelly’s formalism (figure 7) is able to describe the long-time scale evolution of a population (e.g. immune receptors, black line) in response to the known albeit stochastic dynamics of a driving population (e.g. virus, red line). (B) More realistically, other environmental sources (e.g. other viral or bacterial populations, nutrient sources, the emergence of new mutants, orange line) can influence the dynamics of the driving population. (C) Additionally, the evolution of the immune system influences the evolution of the viral population, exerting feedback on its dynamics. This feedback, which is at the heart of the co-evolution problem, makes the dynamics particularly challenging to describe on long timescales.

Considering a stationary viral environment to which the host population is subject (figure 8(A)) is indeed not *a priori* justified. First, with a constant selective pressure to diversify, a limited number of pre-defined set of states is not realistic. A new viral mutant (figure 8(B)) or a new host type *σ* may appear, an event that cannot be described by equation (1) where all possible states *σ* are assumed to be populated at all times (*Nt*(*σ*) ≫ 1). Second, the dynamics of the host population feedbacks on the viral population, thus introducing a non-linear frequency dependence (figure 8(C)). In this case, optimizing the growth rate of the host population is no more justified than optimizing that of the viral population. With no optimization principle, the concept of an optimal strategy that is at the foundation of Kelly’s formalism comes to naught.

### 7.3. Advances in science and technology to meet challenges

We expect advances to come from a convergence of mathematical developments and empirical findings driven by the collection of large scale ecological data. In this respect, the coevolution between bacteria and their viruses (phages) seems to us a particularly promising ecosystem to focus on.

Mathematically, multiple models of interacting populations have been developed, from Lokta–Volterra equations to evolutionary game theory. These models, however, are not obviously connected to the informational models developed for independent environments. Instead, a natural extension of Kelly’s formalism is to optimize the parameters of the viral environment to minimize the growth rate of the host population [59]. This approach assumes, however, a separation of time scales that is to be explained. A more relevant mathematical formalism may be adaptive dynamics [60]. In absence of feedback, invasion fitness, which assesses the capacity of a new mutant to invade a resident population, exactly corresponds to the long-term growth rate of the population of mutants. In the presence of feedback, however, invasion fitness describes only the dynamics on short time scales.

Ecological observations should help direct the development of relevant models. Data on co-existing microorganisms in the oceans shows both extensive diversification and specialization, as well as a lot of diversity within each sub-population [61]. These patterns of evolution, large diversity despite strong selection pressures, are not explained by traditional population genetics. The observed diversity may be transient and formalisms that deal with species extinction need to be employed. Yet, even assuming stable populations, accounting for a feedback of the population onto its environment remains a challenge.

More specifically, data is available on the relative abundances of phages and bacteria in the oceans, which displays a scaling law, albeit with a dependence on depth [62]. Ample genomic data is also available, which reveals a high diversity of gene content with evidence that this diversity is both driven by the interaction with phages and providing a means to adapt to environmental changes [63]. Phages may thus not only be predators but provide a mechanism of regulation that confer on bacterial populations a long-term advantage. As phages are very species-specific, an optimization principle defined at the level of two populations might thus be relevant, which could be cast in Kelly’s formalism.

### 7.4. Concluding remarks

Populations evolve constantly influenced by their environments, and in turn influence changes in their environments. For this reason, the basic concepts of population genetics—mutation, selection, genetic drift, recombination and even ‘population’—are not sufficient to understand the course of evolution. Keeping in mind the importance of timescales for all participating interactions, insights from field data and experiments should help us extend current theoretical formalisms.

### Acknowledgments

This review benefited from work and discussions with Théo Maire.

## 8. How the immune system learns from changing experiences

### 8.1. Status

Evolution is an urge for novelty, because environmental pressures are ever changing. To persist in fluctuating conditions, evolving organisms must adapt to new challenges, without degrading performance of prior tasks. This remarkable ability to generalize manifests at many scales, from biochemical (antibiotic resistance) to ecological (cross immunity). At each scale, it exemplifies an interplay of internal processing and unique experiences—a basic feature of learning. In this perspective, we consider evolutionary learning in the adaptive immune system as a concrete example, where evolution is rapid and learning tasks are complex and changing. But the central questions are general: how does the ability to generalize evolve, given that benefits may only lie in the future? What environmental variations might select for generalization, if specificity more easily evolves in static conditions?

Facing the constant need to fend off novel invaders, jawed vertebrates have developed adaptive immune systems. This mode of protection relies on an extremely diverse and variable repertoire of antigen receptors expressed by B and T lymphocytes in an individual. While T cells do not evolve, B cells produce increasingly higher affinity Abs (secreted B cell receptors) to neutralize pathogens—via a rapid evolutionary process known as AM—and create immune memories.

Theoretical studies have examined adaptive strategies of biological populations to persist in time-varying environments (e.g. [58, 64, 65]). While slow environmental variations appear to favor specialist strategies based on tracking or diversifying (bet hedging), rapid fluctuations may support a non-varying generalist strategy or smear out differences among phenotypes. However, natural environments may change neither too fast nor too slow compared to population response; moreover, these changes are not entirely random. For instance, rapidly adapting pathogens like HIV generate escape mutations on similar timescales to evoked reorganization of the immune repertoire, resulting in an enduring coevolutionary arms race in the host. Amazingly, following the rise and fall of strain-specific responses that chase after successional waves of HIV escape mutants, lineages of bnAbs, capable of neutralizing a vast variety of HIV strains, emerge in a small fraction of individuals [66].

However, these generalist Abs against HIV emerging years into infection never rise to a protective level in any human, struggling to persist even after viruses diversify. In contrast, other highly mutable pathogens, including hepatitis C virus, may go extinct following a faster development of bnAbs. Decades after the discovery of AM by Herman Eisen and Gregory Siskind in 1964, we still lack a complete understanding of what determines the pace, course and outcome of antibody evolution in dynamic environments, which limits our ability to mitigate viral evolution and to accelerate immune control.

### 8.2. Current and future challenges

Recent computational studies have tackled in various contexts how temporally structured environments impact selection of generalists (e.g. [46, 67]). These works stress the importance of not only what and how strong selective forces should be present, but also the time over which they should apply. But the space of possible dynamical protocols (e.g. vaccination strategies) is large and high dimensional: for a small number of examples to enable generalization to novel inputs, what commonality and distinction should be encoded? When will the order of presentation matter? How should one adjust the timescales of variation to the correlations between examples? We need a quantitative framework to provide guiding principles for finding optimal protocols.

While discrete designer environments offer opportunities to enumerate possible scenarios and to obtain a fine view of molecular evolution, natural pathways may display more regular and universal features in continuous and lower-dimensional phenotype space [68]. Yet, current phenotypic models of host–pathogen interaction often treat one or the other as an effective environment, invoking a separation of timescales. This leaves aside two sources of feedback (key to any learning) that may significantly affect evolutionary dynamics and fate: first, mutual feedback between populations evolving on similar timescales leads to fundamentally out-of-equilibrium dynamics and evasion from steady state. These naturally intermediate-timescale variations can no longer be described via effective static environments valid only in the fast or slow limit. Second, ecological processes, such as niche construction, can strongly impact evolutionary modes and phylogenetic patterns. Capturing this interplay requires simultaneous consideration of ecological interactions and evolutionary dynamics in the same framework.

There is also a need for integrative methods in order to shrink the gap between experiment and theory. Immune functions often involve competing needs, such as specificity and generalization, speed and efficacy. Information processing on multiple scales in time and space might complement each other toward a common goal: active patterning of receptors and ligands at cell–cell contacts enables efficient cellular readout of threats; clusters of cells search and compete for antigens distributed in tissues; subdivided B and T cell populations collectively extract antigenic features and encode memories in an organism. This complexity calls for new statistical mechanical descriptions, combined with information measures, to predict emergent phenotypes for comparison with accumulating observations and to suggest new experiments.

### 8.3. Advances in science and technology to meet challenges

To build a quantitative framework to describe dynamic selection of generalists, we need a better understanding of how specialists and generalists are organized in sequence space. On the molecular level, this knowledge relies on high-throughput methods capable of mapping the sequence–function relationship in the mutational neighborhood of target genotypes, i.e., the local fitness landscape. This would allow us to read adaptive dynamics from landscape topography, an idea extended to study long-term adaptation in changing environments [69].

**Figure 9.**
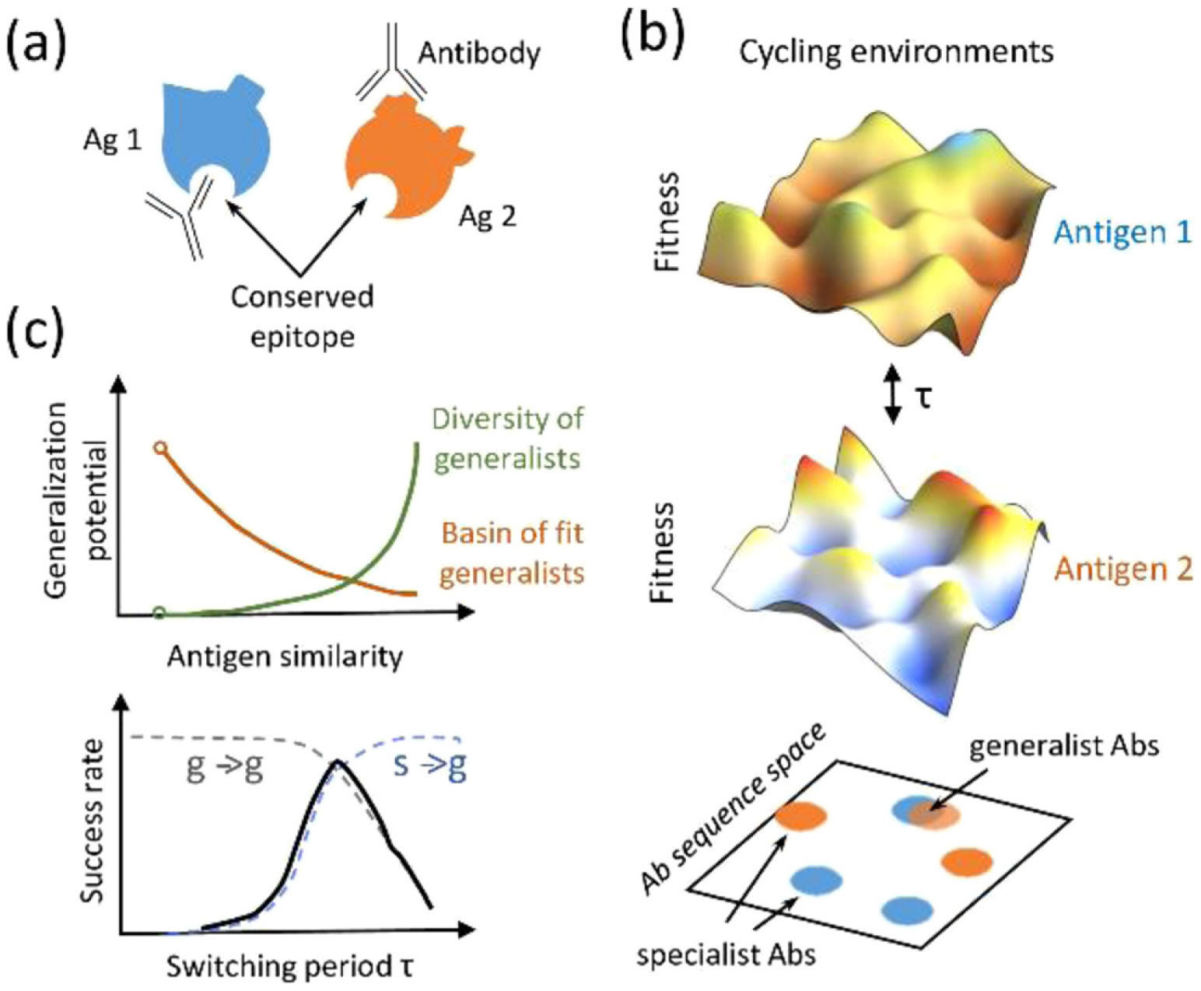
Evolve and maintain generalists via time-varying environments. (a) Generalist Abs recognize the conserved epitope of variant antigens (Ags) while specialist Abs bind well to a particular variable epitope. (b) An illustration of our approach to constructing fitness landscapes that encode how specialists and generalists are organized in Ab sequence space. Each Ag defines a distinct landscape with a distinct set of fitness islands around specialist peaks (blue and orange regions); the generalist peak remains in almost the same location across Ags (overlapping shades). (c) Cycling of sufficiently dissimilar Ags at intermediate timescales can evolve (s → g) and maintain (g → g) generalist Abs unobtainable under very fast or very slow cycling (effectively static environments). Adapted from references [48, 69].

Directed evolution in the lab may test the predicted environmental correlations and timescales that robustly evolve generalists, based on recently developed generative [69] or phenomenological [48] models of evolution in time-varying multi-peaked fitness landscapes (figure 9). In this framework, relative placement of fitness peaks encodes tunable correlations in features across environments. A key insight is that time variations should be slow enough so that appreciable changes can be accumulated to discover generalists, but fast enough to minimize specialization of generalists that have emerged. The existence and range of intermediate timescales depend on the degree and structure of environmental correlations, which potentially offer a way to characterize whether, and where, one would expect non-equilibrium fitness seascapes [70].

Time-resolved measurements of genetic composition along with ecological dynamics are needed to infer phylogenies and account for demographic influence. Corresponding phenotypic studies (e.g. antibody binding and neutralization assays) would allow one to construct low-dimensional theories of eco-evolutionary dynamics, thereby associating phenotypic patterns with population fate. More broadly, we must develop new representations of the sensing and recognition space that captures distinctive properties (e.g. cross-reactivity) and reveals essential learnable features, which are not yet available for immune recognition.

Understanding how information processing in the immune system is integrated across scales counts on interdisciplinary approaches that parallel this goal. Biophysical and physiological measurements are informative of how immune cells sense, distinguish and acquire antigens using energy-consuming active processes. A combination of live imaging and single-cell sequencing would enable simultaneous tracking of cell movement and clonal dynamics in multiple populations. Sampling from different locations in the tissue is needed to study how spatial heterogeneity and connectivity affect the collective response of a population ensemble.

### 8.4. Concluding remarks

Investigating how the immune system learns from changing circumstances presents outstanding opportunities at multiple fronts. Conceptually, it will advance our understanding of truly non-equilibrium regimes of eco-evolutionary dynamics. Such understanding can, in turn, offer novel strategies to alleviate constraints inherent in equilibrium or steady-state conditions. Further, these studies can shed light on information acquisition under dynamic feedbacks, in favor of pathways and outcomes otherwise hard to obtain. General concepts learned will likely generalize to other evolutionary contexts and sensory systems. After all, learning from experiences is much in the timing.

### Acknowledgments

SW gratefully acknowledges funding from the Dean of Physical Sciences and the Bhaumik Institute for Theoretical Physics at UCLA.

## 9. Time-dependent drug sequences for slowing antibiotic resistance

### 9.1. Status

Antibiotic resistance is a growing threat to public health. Bacteria exploit a diverse store of genetic and phenotypic defences to counter antibiotics, and the rapid pace of microbial adaptation makes the long-sought goal of ‘resistance-proof’ drugs appear increasingly unlikely. As a result, there is significant interest in developing evolution-based strategies for slowing, or reversing, resistance by judiciously applying currently available drugs. One possible approach is to use multiple-component therapies, which force bacteria to adapt to antibiotics targeting different cellular processes. The simultaneous use of multiple drugs is particularly promising because the effects of the drugs are often coupled so that each drug either strengthens or counteracts the other. These *drug interactions* have the potential to enhance therapies by partially decoupling the short-term inhibitory effects of drugs from their propensity to select for resistance [71]. A second strategy is to use multiple drugs in sequence, forcing bacteria to adapt to time-varying environments. These *antibiotic cycling* approaches have so far achieved mixed results, particularly when applied at the hospital level [74]. However, recent studies have reinvigorated interest in temporal strategies based on *collateral sensitivity* (CS), an evolutionary ‘side-effect’ of acquired resistance [72, 73, 75-78]. CS occurs when a population evolves resistance to one drug (the ‘selecting drug’) while simultaneously exhibiting increased sensitivity to a different drug [79]. CS appears to be ubiquitous—at least *in vitro*—underscoring the notion that pathways for adaptation to different drugs, even those from different classes, are inextricably linked. In the context of drug cycling, these collateral effects couple adaptation at different time points, potentially forcing cells into a time-dependent version of multi-task optimization. Cells exposed to dynamic environments are known to adopt phenotypic or genetic strategies to exploit statistical features of the changing environment. The question, for antibiotic cycling, is in some sense just the opposite: is it possible, by systematically manipulating the environment over time, to steer evolution toward a desired state? More specifically, can we use particular sequences of drugs to slow resistance by harnessing the correlations that link resistance levels to different antibiotics?

### 9.2. Current and future challenges

There are many practical obstacles to overcome before new multi-drug strategies can be deployed in the clinic. The barriers range from technological (optimized approaches may require, for example, new tools for rapid diagnostics) to translational (e.g. validation of *in vitro* approaches in patient samples and animal models) and even economical. In addition to these practical hurdles, there are a number of conceptual challenges to designing drug sequences that potentially limit resistance evolution. These challenges represent open basic science questions that potentially link fundamental concepts from evolutionary biology to an eminently practical issue in medicine and public health.

First, the molecular mechanisms of CS are often unknown, meaning that collateral sensitivities are difficult to predict and must be identified empirically [75]. In addition, experiments suggest that collateral effects often appear stochastic—that is, adaptation in replicate populations frequently leads to different phenotypic profiles of collateral resistance, even when starting from a common ancestral strain [73, 76, 77]. Furthermore, collateral effects are not limited to sensitivity; indeed, collateral resistance (often termed ‘cross-resistance’) is common and could limit the utility of simple cycling strategies. Phenotypic profiling in multiple species also indicates that collateral effects are often asymmetric—for example, adaptation to drug 1 may lead to increased sensitivity to drug 2, while adaptation to drug 2 leads to increased resistance to drug 1—and they may depend sensitively on the genetic background—and hence, the adaptation history—of a given population [76, 77]. The picture that emerges is enticing, as collateral effects offer a new dimension for systematically tuning multi-component therapies. Yet despite the apparent ubiquity of CS, designing drug sequences that exploit these correlations to slow evolution is an ongoing challenge.

### 9.3. Advances in science and technology to meet challenges

**Figure 10.**
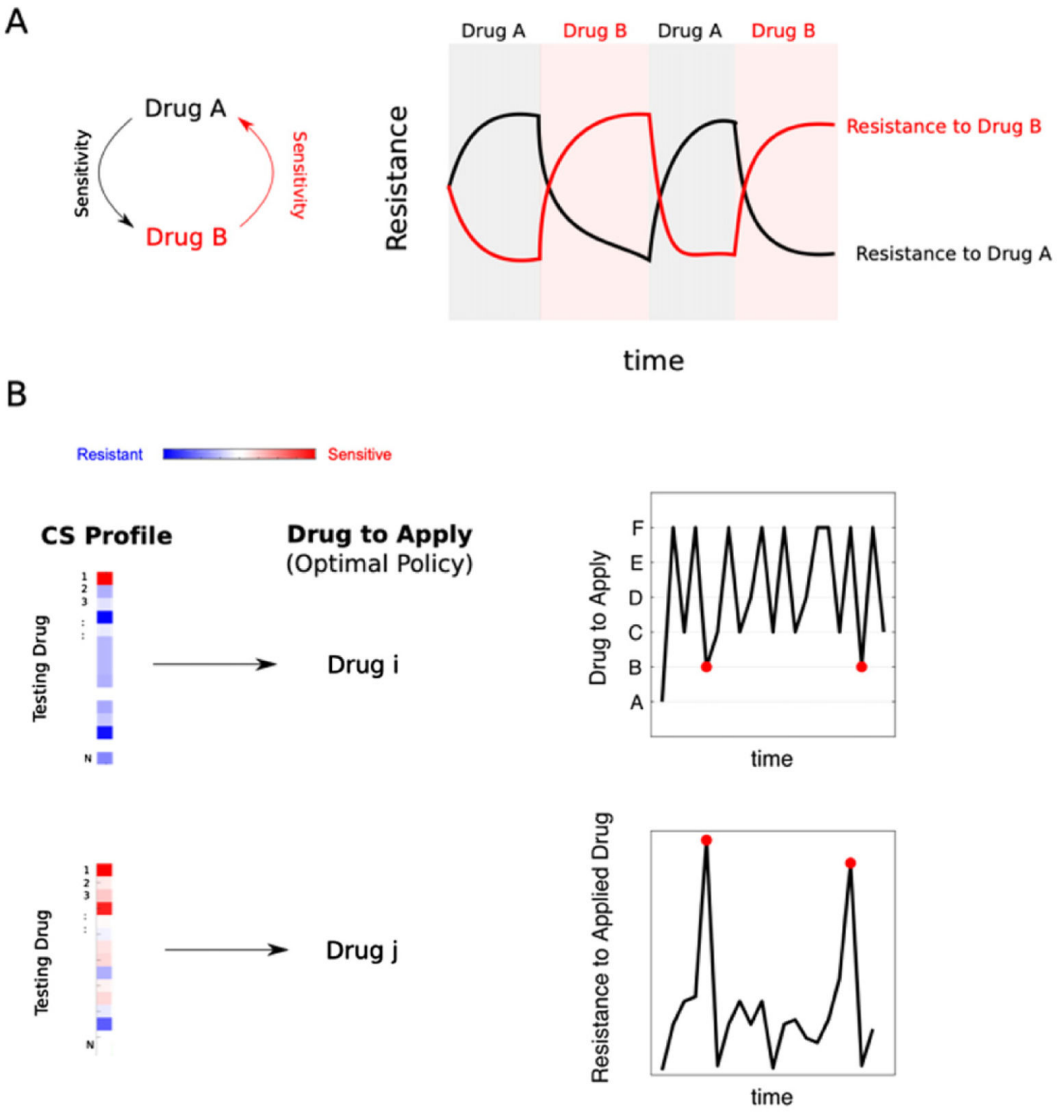
Time-varying drug sequences for slowing the evolution of resistance. (A) Drugs A and B induce reciprocal CS: adaptation to A increases sensitivity to B, and adaptation to B increases sensitivity to A. Right panel: schematic showing resistance to each drug (A, black; B, red) over time during the cyclic application of drugs A and B (see [71, 72]). (B) Left panel: an optimal drug policy uses stochastic control algorithms to assign each CS profile (i.e. a set of values that define the resistance of the population to each of *N* testing drugs) to a single applied drug. Policies designed to minimize long-term resistance generate aperiodic drug sequences (top right). These optimized sequences correspond to frequent periods of low resistance interspersed with rare periods of high resistance (red dots). The drugs corresponding to periods of high resistance (in this case, drug B) provide little instantaneous inhibition but steer the population to a more vulnerable future state (see [73]).

The increased interest in CS was sparked, in part, by innovative work aimed at identifying ‘CS cycles’—periodic sequences of antibiotics in which the drug applied at one timestep is expected to induce sensitivity to the drug applied at the next step [72] (figure 10(A)). Network analysis of empirical CS profiles identified hundreds of potential cycles, many involving three or more drugs. In addition, switching between two drugs can also lead to transient changes in resistance levels, a type of phenotypic memory recently termed ‘cellular hysteresis’ [80]. Taken together, these results suggest that time-varying drug sequences may be sufficiently flexible to slow resistance while allowing for fine-tuning of additional clinical or practical objectives.

However, identifying optimized drug sequences is complicated by the variability in CS profiles, which can differ across species and even replicate-to-replicate, in part because of the stochastic nature of adaptation on the underlying fitness landscapes [73, 76]. Despite these challenges, recent findings in both the laboratory and clinic point to some measure of stability in the evolutionary dynamics [77, 78], offering hope that there are actionable trends buried beneath the complexity. One option for exploiting these trends is the use of likelihood scores, which place evolutionary therapies in a probabilistic framework [76].

In a similar spirit, recent work from our group drew on theoretical tools from stochastic control to design optical drug sequences for slowing resistance [73]. To do so, we derived data-driven optimal drug policies that assign a single drug to every possible CS profile (figure 10(B)). Drug sequences based on these profiles reduced growth and slowed adaptation in lab evolution experiments, outperforming treatments using single drugs or small (*N* = 2, 3) cycles. The approach revealed a new conceptual strategy for slowing resistance by interspersing frequent steps of instantly effective drugs—which provide short-term inhibition of pathogen growth—with rare steps of relatively ineffective drugs, which shepherd the population to a more vulnerable future state.

### 9.4. Concluding remarks

Given the slow pace of drug development and the seemingly infinite adaptive capacity of bacteria, antibiotic resistance is likely to pose an increasing threat to public health in the decades ahead. There are no ‘magic bullet’ solutions on the horizon, and the battle against resistance calls for innovative strategies spanning multiple disciplines and a range of length scales, from the molecular scale to the level of hospitals and communities. Translating new approaches to the clinic will require much additional work, including an improved understanding of the evolutionary forces that shape adaptation in human hosts. Nevertheless, recent progress offers hope that multi-drug sequences may one day form the basis of therapies designed to exploit time-dependent evolutionary trade-offs of drug adaptation.

## 10. Memory and adaptation in time varying environments

### 10.1. Status

Quantitative studies of cellular physiology have led to new understanding of growth and size control mechanisms, including general principles that operate in proliferating populations [81-83]. In contrast, little is known about how such mechanisms evolved, for example (i) whether there exist specific environmental pressures that select for the observed growth modes, (ii) whether growth control mechanisms optimize some cost/benefit tradeoff and if so over what timescales, and (iii) how do genetic networks evolve to achieve robust growth and how are they maintained over evolutionary timescales.

Long-term laboratory evolution experiments have provided illuminating, and often surprising, insights on how evolution works in practice [84], but were not designed to answer the types of questions outlined above. In particular, gene regulatory networks enable survival in time-varying environments, thus studying the relevant principles requires both the experimental capacity to measure growth under controlled, external fluctuations, as well as new theory that will provide a framework for designing powerful experiments.

**Figure 11.**
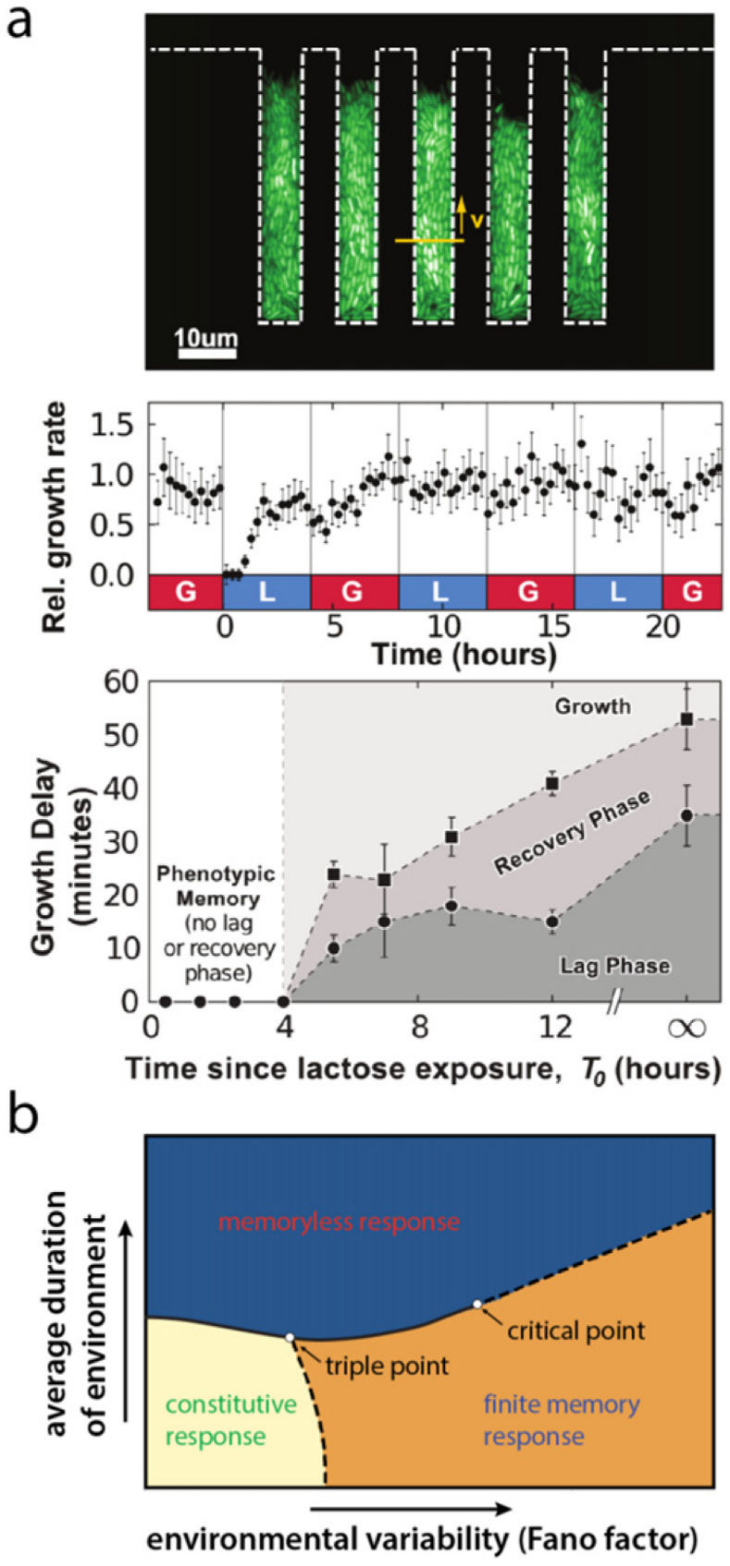
(a) The *chemoflux* microfluidic device, in which growth of a population of cells contained in 10 *μ*m wide chambers is continuously monitored. By flowing different media through a main flow channel, the environment inside the chambers can be quickly exchanged from one nutrient type to another, or from a condition of stress to growth. Data represents a typical response to fluctuating sugars glucose (G) and lactose (L). In the first fluctuation, cells go through a lag phase, followed by recovery to exponential growth. During the lag phase cells produce and accumulate metabolic proteins. Upon repeated fluctuations the lag phase disappears. This effect has been explained through inheritance of very long-lived *lac* proteins, which provide the physiological memory of the cell. (Adapted from [86]). (b) The phase diagram of optimal response strategies in fluctuating environments. Strategies are separated by solid (dashed) curves corresponding to first order (continuous) evolutionary phase transitions, with their intersections shown at the triple point and a critical point. A finite memory response is optimal exclusively under random fluctuations. (Adapted from [88]).

Recent technological advances [85] have shown new and promising insights into how cells have evolved their responses to changes in their environments. In fluctuating environments (figure 11), bacteria utilize physiological memory as a way to minimize metabolic [86] and antibiotic stress [87]. These experiments coupled with theoretical work, can determine the environmental conditions in which such memory is beneficial for cells, and how such memory can evolve.

This approach has led to predictive theories that establish optimal response strategies for survival under fluctuating stress, depending on how variable or unpredictable is the environment [88]. By analogy to condensed matter systems, the space of optimal responses can be represented in a phase diagram (figure 11(b)) where different strategies are separated by continuous and first order evolutionary phase transitions, and which correspond to different ways of evolving the optimal response in a gene circuit.

### 10.2. Current and future challenges

Such approaches can also be used to study the emergence and spread of antibiotic resistance across a population of bacteria, a pressing public health concern and a major challenge in the field of evolutionary biology.

**Figure 12.**
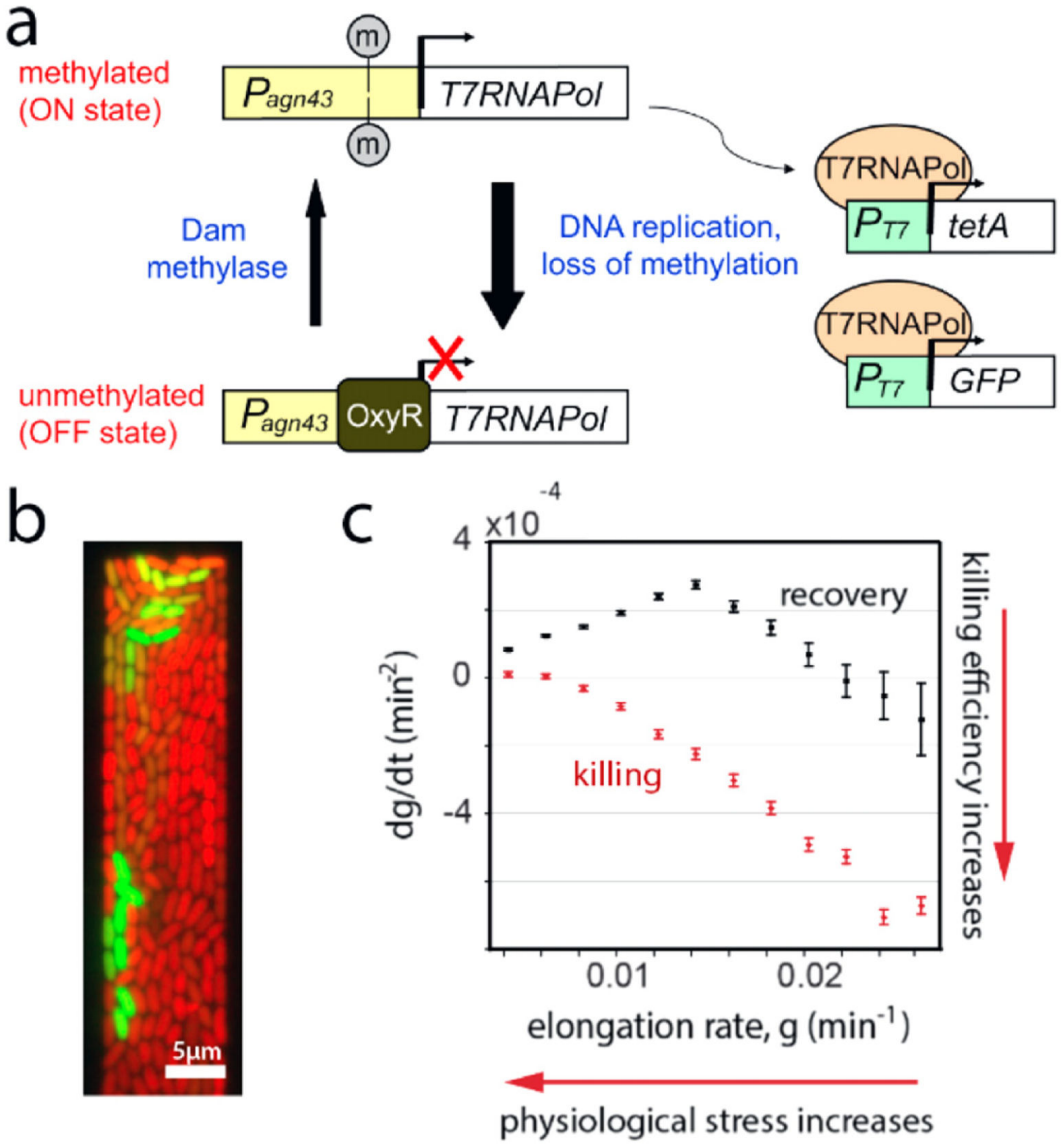
The agn43 promoter driving the T7 RNA polymerase stochastically generates tetracycline-resistant cells, switching epigenetically between transcriptional ON and OFF states, with rate 0.1%–1% per cell division (panel a), which is visualized by a GFP reporter (panel b) [87, 89]. Single cell measurements of elongation rate dynamics (panel c) show dependence of killing efficiency on physiological stress response. (Adapted from [87].)

Cellular response to fluctuating antibiotic stress, where a period of growth is followed by a period of exposure to tetracycline, a bacteriostatic antibiotic, shows how the metabolic state of a cell affects its ability to evolve resistance (figure 12) [87]. During tetracycline pulses, cells exhibit strongly reduced cell division and elongation rates. Once tetracycline is removed, cells slowly recover, which is reflected in the increased rate of elongation. The cells’ elongation rate thus provides an instantaneous readout of cellular stress. Remarkably, for low elongation rates the treatment is ineffective, indicating that the cellular stress response that halts cell growth confers physiological protection against the antibiotic. While treatment is effective for high elongation rates, bacterial cells undergoing treatment are rarely found in such rapidly proliferating states. A more realistic scenario lies between the two extremes, at intermediate elongation rates.

Surprisingly, however, cells recover fastest at intermediate physiological stress, rendering a large range of treatments not only ineffective, but also allowing cells ample time to acquire resistance. Physiological memory, over which cells time-average the antibiotic dose, strongly impacts the elongation rate dynamics and thereby influences rate at which resistance emerges. A more detailed understanding of these processes and how they contribute to the evolution of resistance could lead to more successful strategies for mitigating or reversing the spread of antibiotic resistance.

### 10.3. Advances in science and technology to meet challenges

Memory is a basic principle that governs the behavior of single cells in changing environments across a wide range of timescales. To move along chemical gradients, *E. coli* use memory of their previous sensory inputs in a feedback that regulates their flagellar motor. Bistable switches can induce distinct stable expression states that can persist for many generations. In fluctuating environments, cells utilize physiological memory to reduce metabolic and antibiotic stress. The examples in figures 11 and 12 show that cost-benefit tradeoffs, which classically are assessed at the level of single cell metabolism, need to be accounted for throughout individual cells’ histories [83], across a heterogenous population [87], and between distinct populations that experience different time-varying environments [88, 90].

Current experimental methods enable all of the above measurements and analyses, and in fact greatly surpass existing theoretical understanding of these biological processes. Due to the complexity of biological systems, it remains challenging to recognize the general principles that determine which strategies will evolve and how changes in the environment drive the evolution of cellular physiology and impact population heterogeneity. Therefore, new theories—that span from individual behaviors to heterogeneous populations to larger-scale ecological structures—are needed to provide strong predictions, while new experimental designs are required to isolate and test those ideas in model systems.

### 10.4. Concluding remarks

In this article we highlighted the advantage of studying synthetic biological systems in the lab as a testing ground for the development of evolutionary theory. The ability to precisely control and fluctuate the environment, in combination with synthetic biology, can enable rigorous, quantitative testing of theoretical predictions. Bacteria exhibit a wide range of behaviors, including memory, responsiveness, sensing, and stochasticity, each of which have distinct benefits in time varying environments. By perturbing these mechanisms experimentally, it may be possible to study some of the general principles of evolution at lab accessible timescales, and to test new theoretical approaches that are sufficiently powerful to enable evolutionary predictions over longer timescales.

## 11. The origins of allostery

### 11.1. Status

Allostery—the functional coupling of distantly positioned amino acid residues in proteins—plays a key role in nearly all cellular processes. In different manifestations, it represents information flow within and between proteins, control of protein activities through regulatory modifications, and cooperativity in oligomeric assemblies [91]. For these reasons, a significant body of work has been focused on elucidating the phenomenological, mechanistic, and generative principles of protein allostery. The goal is to develop models for the physics of long-range intramolecular couplings within proteins consistent with their evolutionary origin.

However, the problem has been a difficult one. Understanding allostery in any general sense starts with defining the pattern of energetic interactions between all amino acids in a protein in all relevant configurational states. This pattern specifies both the structure (the mean position of all atoms) and its dynamics (fluctuations, both independent and collective) over the evolutionarily selected reaction coordinate. But, proteins are held together by an extraordinarily subtle balance of forces that produce marginally stable structures [92], and deducing the net value of residue interactions remains a challenge for experimental and computational approaches. An added complication is that long-range couplings fundamentally arise from the nonlinear (epistatic) interactions of amino acid residues [91, 93]. The full theoretical combinatorial complexity of such interactions is inaccessible to any scale of experimental analysis.

**Figure 13.**
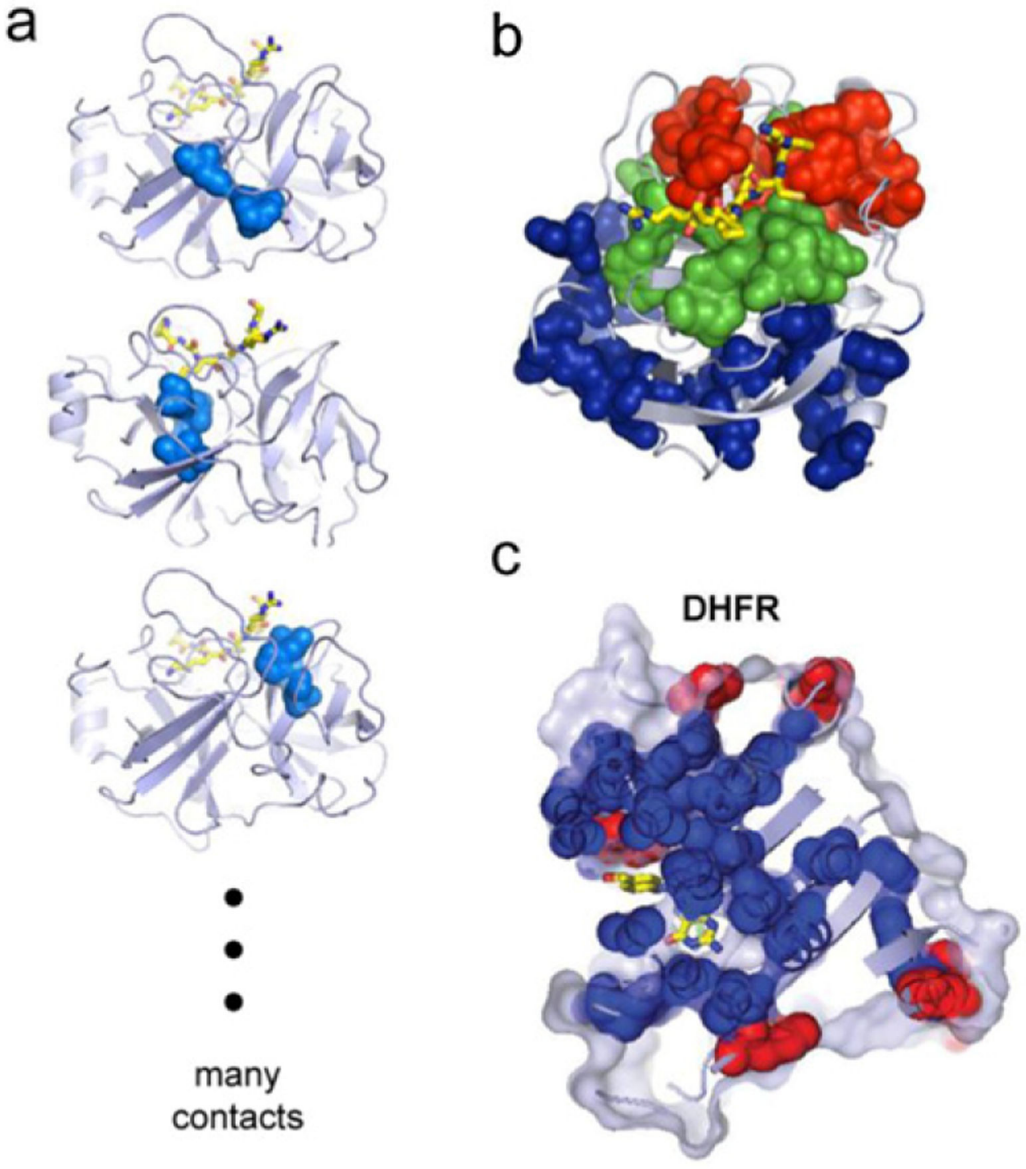
(a) Examples of pairwise coevolving amino acids (blue) in the S1A family of serine proteases, comprising direct contacts in the tertiary structure. Data are shown on the structure of rat trypsin (PDB 3TGI). (b) Three sectors (red, green, and blue) in the S1A family, demonstrating physically connected coevolving networks within the structure. (c) A sector (blue spheres) in DHFR (PDB 1RX2), connecting the active site (marked by bound substrate, yellow stick bonds) to several distant surface sites (marked in red).

In recent years, a different approach to understand allostery has been to leverage the growing databases of protein sequences to make statistical models for the pattern of amino acid interactions. The simple idea is that evolution has been mutating and selecting proteins for a long time and given enough samples of extant sequences that have survived this process, we might be able to infer the relevant interactions between amino acids by measuring the correlated evolution of those positions [94-96]. This ‘statistical genomics’ approach has revealed two qualitatively different kinds of amino acid interactions within proteins: (1) a large number of coevolving pairs of amino acids (~*L*/2, where *L* is the length of the protein sequence) and (2) a few (1–3, in work to date) collectively evolving groups of amino acids, called ‘sectors’. The coevolving pairs often correspond to direct contacts in protein tertiary structures (figure 13(a)), while sectors correspond to more distributed networks of amino acids that connect primary functional sites of proteins to a few distantly positioned surface sites (figures 13(b) and (c)). Sectors correspond to known allosteric mechanisms [97], have predicted previously unknown allostery in proteins [96], and have been used to engineer allosteric regulation in proteins [98].

It is important to say that like any purely phenomenological analysis, the pattern of coevolution provides no intrinsic information about either the physical mechanism of allostery or its origins in the course of evolution. Nevertheless, the statistical genomics approaches provide models for the global pattern of amino acid interactions that motivate new experiments to probe mechanisms of allostery. This work is ongoing and may help to better understand the physics of long-range couplings in proteins.

### 11.2. Current and future challenges

But, how does allostery arise in evolution? The finding that coevolving networks of amino acids (sectors) correspond to regulatory mechanisms would itself seem to imply *a causal principle of origin*. That is, if sectors mediate allosteric regulation, it is perhaps natural to think that they arose in evolution due to selection for the regulatory function. However, further considerations cast serious doubts about this possibility. First, there is the finding that sectors also occur in proteins in which there is no evidence for allosteric communication or regulation. For example, in the metabolic enzyme dihydrofolate reductase (DHFR), the sector connects the active site to a specific subset of surface positions distributed widely throughout the tertiary structure (figure 13(c)). Experimental studies confirm that these sites are indeed capable of supporting allostery in the sense that engineered regulatory inputs at those sites can selectively control enzyme activity [98]. However, DHFR has no known natural allosteric regulators and no role in signal transmission.

It is possible to sweep this problem under the proverbial rug by simply asserting that these sites reflect yet undiscovered regulatory mechanisms or reflect residual constraints from a past history of allosteric regulation. However, further complications arise from considering the evolutionary process that would be necessary to build allosteric networks within proteins if their origin lies in selection for regulation. Allostery implies epistasis along evolutionary trajectories, such that the effect of mutation at one site in an allosteric network is conditional on mutations at other positions. For long-range allostery, the capacity of a distant surface site to control the primary functional site will depend on a long series of conditional mutations, with no guarantee that intermediates along the path can support regulation. In such a scenario, it is entirely unclear how allostery could originate as a causal result of direct selection for regulation.

It is also possible to sweep this problem under the rug by invoking global forms of epistasis in which every perturbation in a protein influences every other position, but it is clear that this represents only a special case. What then is a plausible model for the origin of allostery in proteins? Two lines of work have now supplied potential answers.

The first comes from recognizing that natural proteins are constrained by not just the physics of folding and function but also the need to adapt as conditions of selection fluctuate in the environment. In principle, *adaptability* places unique constraints on the pattern of amino acid interactions. One such constraint is functional connectivity, meaning that all intermediates along an adaptive path must maintain function above a selection threshold [99]. Recent studies in the PDZ domain, a protein interaction module, show that this constraint depends on the availability of a special class of mutations called ‘conditionally neutral (CN)’ [100]. Such mutations are neutral for the current condition of selection (and therefore can accumulate as standing genetic variation in populations) but display significant gain-of-function in new conditions of selection. CN enhances adaptation by essentially uncoupling the generation of phenotypic diversity from the need for that diversity. Thus, when selection pressures randomly vary, pre-existing CN mutations in the population can initiate a path of adaptation.

**Figure 14.**
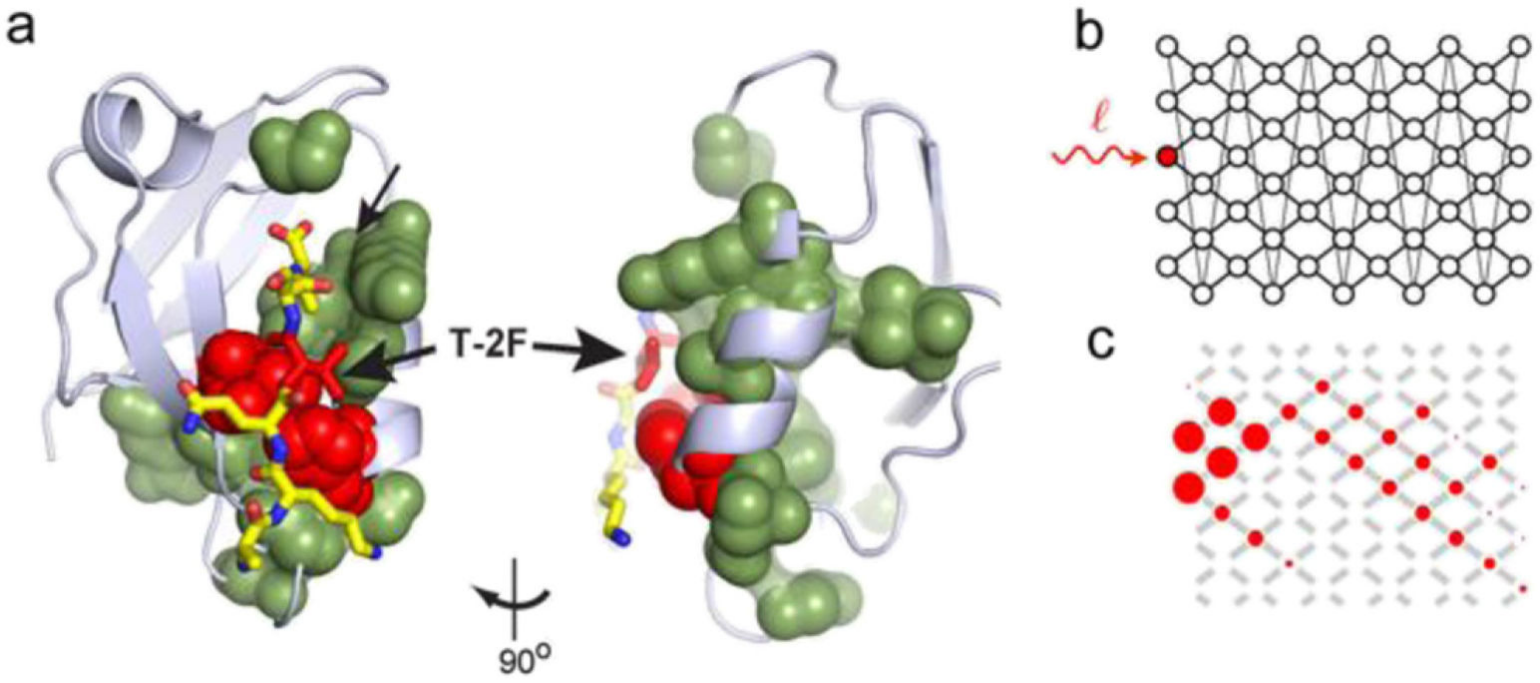
(a) The spatial distribution of adaptive mutations in the PDZ domain in response to a class-switching T–2F mutation in the ligand (shown as stick bonds); green spheres mark positions harboring CN mutations. Two views of the domain (PDB 1BE9) are shown, demonstrating that CN mutations exclusively occur along allosteric networks extending through the protein structure. (b) A two-dimensional spin lattice model for a protein, with the ‘ligand’ represented by an external field acting at a single boundary node (the ‘binding site’). (c) Evolving the parameters of the spin model under conditions where the lattice must discriminate a right ligand from a wrong ligand produces a final model in which the right ligand specifically triggers an allosteric conformational change through the model protein (red nodes, with the size of the node indicating the strength of the conformational change). Data in (b) and (c) are from reference [101].

With regard to allostery, the key finding is that all CN mutations in proteins are allosteric and intimately associated with sectors (figure 14(a)) [100]. Thus, in addition to functional regulation, another role for allostery (and protein sectors) is the capacity to adapt. Turned around, this logically implies that the origin of allostery in proteins could be simply in the need to adapt to fluctuating conditions of selection. In this model, allosteric networks get built in evolution not by direct selection for regulation, but because they generate CN mutants that facilitate adaptation in time-varying environments. The use of allosteric networks for signaling or regulation is then a derivative of this more fundamental process. A key prediction of this model is that the existence, architecture, and conservation of allosteric networks depends fundamentally on the nature and temporal structure of environmental fluctuations in conditions of selection.

A distinct but non-exclusive model for the origin of allostery has also been recently proposed. Using simplified lattice models of proteins, the proposal is that allostery can also evolve in proteins as a natural solution to the problem of achieving specificity in molecular recognition (figures 14(b) and (c)) [101]. The intuitive explanation is that specificity requires fine control over energetic states to distinguish right and wrong substrates at protein functional sites, and that such fine control is statistically more likely from allosteric networks rather than from orthosteric positions alone.

In general, both adaptability to time-varying environments and binding specificity could collaborate to produce and sustain allosteric networks within proteins. The salient point is that in both cases, allostery emerges in evolution purely as a constraint acting on the primary functional site of proteins. Once built, this architecture can then be co-opted to enable signaling, regulation, and other classical manifestations of allostery. This represents a model for the evolution of allostery that is consistent with a Darwinian process of stepwise variation and selection.

### 11.3. Advances in science and technology to meet challenges

How can we test these new models for the origin of allostery in proteins? The key is to carry out forward evolution of protein molecules with full control over the essential parameters—mutation rate, population size, and the statistics of applied selective pressures. If this can be achieved, the idea is to conduct many independent trajectories of evolution while selecting proteins for binding affinity or specificity in constant or time-varying conditions of fitness. With appropriate controls, deep sequencing of these trajectories and deep mutational scanning of evolved proteins can provide estimates of both the standing variation and the pattern of allostery. In principle, such experiments could provide a rigorous test of the necessity and sufficiency of selection for specificity and/or adaptability to sustain allosteric networks in proteins without direct selection for regulation.

Are such forward evolution studies feasible? In recent years, powerful experimental methods for rapid, continuous, automated forward evolution of proteins have been reported [102, 103]. With further developments to enable continuous evolution for binding specificity and adaptability, it should be possible to carry out the required studies. A complementary approach is to extend the network models for protein stability and function [101] to computationally probe the role of binding specificity and/or evolution under fluctuating environments in shaping patterns of allostery. The combination of experiments in real proteins and computational ‘toy’ models may represent a powerful strategy to converge on minimal models for the origin of allostery.

### 11.4. Concluding remarks

Models for biological systems must go beyond just explaining mechanism to provide a description of their origin through the process of evolution. In general, this means understanding how functional constraints at the current moment collaborate with adaptation in time-varying environments to specify the design of biological systems. For the classical problem of allostery, the hope is that the new ideas and emerging technologies described here can help produce generative models for long-range intramolecular couplings within proteins.

### Acknowledgments

I thank Olivier Rivoire, Arvind Murugan, and members of the Ranganathan lab for discussions and comments. RR acknowledges support from NIH Grant RO1GM12345, a Data Science Discovery award from the University of Chicago, and the Center for Physics of Evolving Systems at the University of Chicago.

## 12. Mechanisms of rapid evolution

### 12.1. Status

Evolution is increasingly being recognized as a central component of two of the most urgent societal problems [104]. While the physics of global climate change is relatively well-understood, the response of the biosphere is harder to predict, especially the extent to which microbial evolution can influence the feedback between soil and marine systems to the carbon and other biogeochemical cycles [105-107]. Even the sign of this feedback effect is hard to assess. The emerging world-wide health crisis due to the unexpectedly rapid evolution and proliferation of antibiotic resistant strains of pathogenic bacteria is our second example, one that underscores how imperfectly we understand the mechanisms of evolution [108]. In fact, it is even the case that climate change can accentuate the problem of antibiotic resistance [104].

Evolution is often thought of as the product of two independent classes of process: (1) the generation of mutations; (2) the dynamics within a fixed environment that selects and ultimately conveys genetic variation to fixation or dominance in a population. This narrative assumes a separation of timescales between (1) and (2) but neglects the fact that many ecosystems, especially those with microbes, show rapid genetic adaptivity through strong selective stress arising either from environmental conditions or antagonistic predation [109, 110]. The resulting phenotypic diversity [109] contains individuals with new traits that in some cases have been documented to further induce new links or forms of interaction with others [111]. For a constant driving force, either a chemical potential difference across the ecosystem, or a constant flux of energy, the resulting long time dynamics is an ecosystem in a non-equilibrium steady state [111], characterized by constant change and the generation of new niches [112], as opposed to one that is in a static equilibrium steady state, characterized by a fixed community structure. In other systems, such as methanogenic bioreactors [113] and the global ocean microbiome [114], it is known that there is a non-equilibrium steady state, characterized by a constant production rate, but constant taxonomic turnover, suggesting the emergence of a collective metabolism for the community.

Whether or not there is a phase transition between these two classes of stationary states as a function of driving force is an interesting but unresolved fundamental question. When the interactions between ecology and evolution are strong enough, such that the evolutionary timescale is comparable to the ecological timescale, qualitatively new phenomena arise: rapid and successive emergence of evolved traits interfere with the ecosystem, resulting in significant changes in population dynamics and spatiotemporal patterns.

The purpose of this roadmap article is to draw attention to two recent highly simplified examples of these phenomena, which are sometimes called *rapid evolution*. The first focuses primarily on population dynamics: anomalies in population cycles can reflect the influence of strong selection and the interplay with mutations (standing variation or de novo). The second focuses primarily on the way in which ecological structure can potentially be influenced by what is arguably the most powerful source of genetic novelty: horizontal gene transfer (HGT). Our understanding of the role of HGT in shaping ecosystems, and vice versa, is in its infancy, but we now have the tools to begin to not only understand these phenomena but to ask the pertinent question of how one manages such dynamic ecosystems. It is well-documented that HGT as well as population flow is central to the antibiotic resistance crisis [108] and one would expect that it plays a role in the biological response to climate change. To achieve a full understanding of rapid evolution in all its manifestations will require a concerted experimental and theoretical effort.

### 12.2. Anomalous population dynamics in rapid evolution

The first example focuses on anomalous population dynamics due to rapid evolution. The anomalous dynamics is characterized by abnormal phase relationships and periodicity in population cycles. Certain predator–prey ecosystems systems, such as rotifer–algae [115] and phage–bacteria ecosystems [116] , exhibit a *π* phase difference between the time series of predator and prey populations, together with a longer period for their population cycle, as opposed to the typical predator–prey phase difference of *π*/2. This abnormal phase difference is associated with the emergence of a mutant prey which has a defense against the predator but at some metabolic cost (so-called *evolutionary cycle*). What is more bizarre is that in some systems, following a mutation, the phase difference disappears as the prey population becomes almost constant in time while the predator population still oscillates but with a longer period than before the mutation arose (the so-called *cryptic cycle*). Whether or not the evolutionary cycle or cryptic cycle occurs depends on the metabolic cost of defense [117].

**Figure 15.**
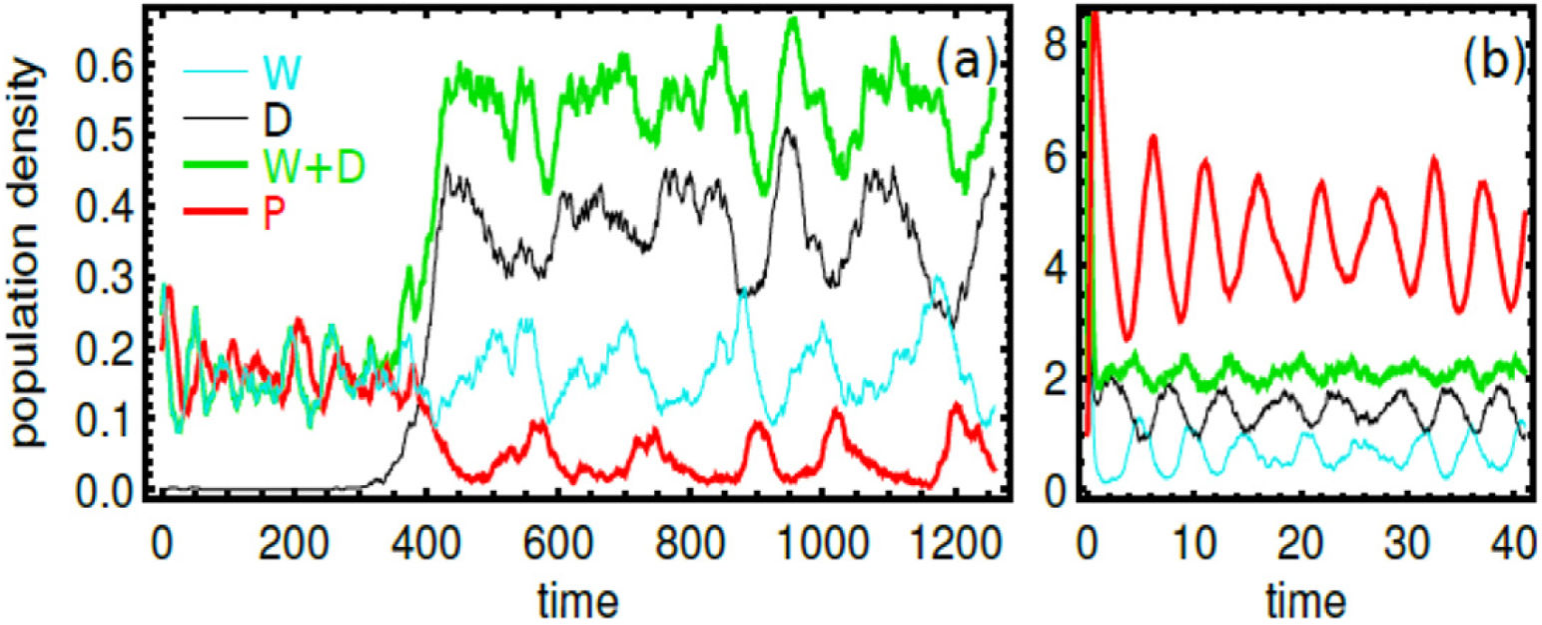
Rapid evolution individual-level simulations capture the eco-evolutionary dynamics characterized by anomalous phase relationship in the experiments, e.g. (a) a transition from the conventional *π*/2 phase difference to the out-of-phase oscillations between predator (red) and total prey (green) densities and (b) a case with a constant total prey density [117].

Due to the necessarily small numbers of mutants at least during the initial stages of these processes, one must properly take into account the discreteness of populations and their spatial extent [118]. The mathematical tools to do this properly use stochastic individual-level models to describe the interactions between members of the ecosystem, and statistical mechanics techniques to deduce the resulting dynamics at the population level [119, 120]. By including the trade-off between selection on reproduction and the metabolic cost of defense against predation, a minimal stochastic individual-level model [117] reproduces the rapid evolution that was found in chemostat experiments of rotifer–algae [115] and phage–bacteria ecosystems [116]. Under strong predation selection, a defended prey can arise from mutation, causing the population dynamics to transition from the normal cycle with a *π*/2 phase shift to the evolutionary cycle with a *π* phase shift between the predator and the total prey. The additional *π*/2 phase delay comes from the fact that the wild-type prey, which is mostly consumed by the predator, can only grow back after the defended sub-population starts to decrease due to the depletion of food. When the metabolic cost of the defended prey is low enough, the regrowth of the wild-type prey is delayed more. If the delay is so great that the wild-type prey can only resume growth after the defended prey population has decreased sufficiently, the phase delay of the wild-type prey behind the defensive prey can become *π* so that the total prey population looks almost constant with time, leading to a cryptic cycle. The individual-level model shows that the anomalous dynamics can arise from demographic noise in rapid evolution, without special assumptions or fine tuning (figure 15). Deterministic models are problematic, as they cannot even capture regular population cycles in a qualitative way without introducing phenomena extra to the Lotka–Volterra description, such as functional response [119]. So far, we have focused primarily on well-mixed systems. However, in practice, one may be interested in invasion fronts, regime shifts or range expansion. In these cases, the need for correct treatment of demographic stochasticity is even greater, because of the presence of fronts where the populations are necessarily small. The study of the potentially interesting spatio-temporal patterns [120] forming in rapid evolution is a rich topic for future work.

### 12.3. Collective rapid evolution

Our second example is from marine microbial ecology, and involves a case where spatio-temporal dynamics emerges from the eco-evolutionary feedback at various scales. In such cases, different evolutionary mechanisms intertwine and lead to scale-dependent feedback, manifested by coevolution from genetic variations, spatio-temporal population dynamics and spatially-varying selection pressure from the environment.

We will focus on a phage–microbe ecosystem, which is usually modeled simply through Lotka–Volterra dynamics. However, in the microbial world, ecological relationships are more complicated than this due to rapid evolution at the genomic scale. In fact, it seems that phage are multifunctional: now only do they exert predation pressure that reduces the bacteria population, but they also transfer genes that can help increase the bacteria population. The way in which this happens in detail is a possible instance of multi-level selection: at the level of the individual bacteria, phage attack is a strong selection pressure. But at the level of the community, there is an emergent fitness benefit which allows the population to grow and even expand its range.

A remarkable example showing the significance of HGT-involved multiscale feedback as a driving force for evolutionary complexity and stability is the most-abundant phototropic organism, *Prochlorococcus spp.* [61]. This marine cyanobacterium experiences predation from cyanophages that, surprisingly, were found to carry photosynthesis genes. Phylogenetic study showed that these genes had been horizontally transferred first from cyanobacteria to cyanophages and back and forth multiple times [121]. Interactions with cyanophage are assumed to be important for the evolutionary pattern and diversity of *Prochlorococcus*. Specifically, *Prochlorococcus* exhibits niche stratification of two dominant ecotypes: the high light-adapted ecotype near the sea surface evolved 150 million years ago from the ancestral low light-adapted ecotype at the lower sea level. Due to the depth-dependent absorption spectrum of light, the different ecotypes utilize distinct light intensities and spectra.

**Figure 16.**
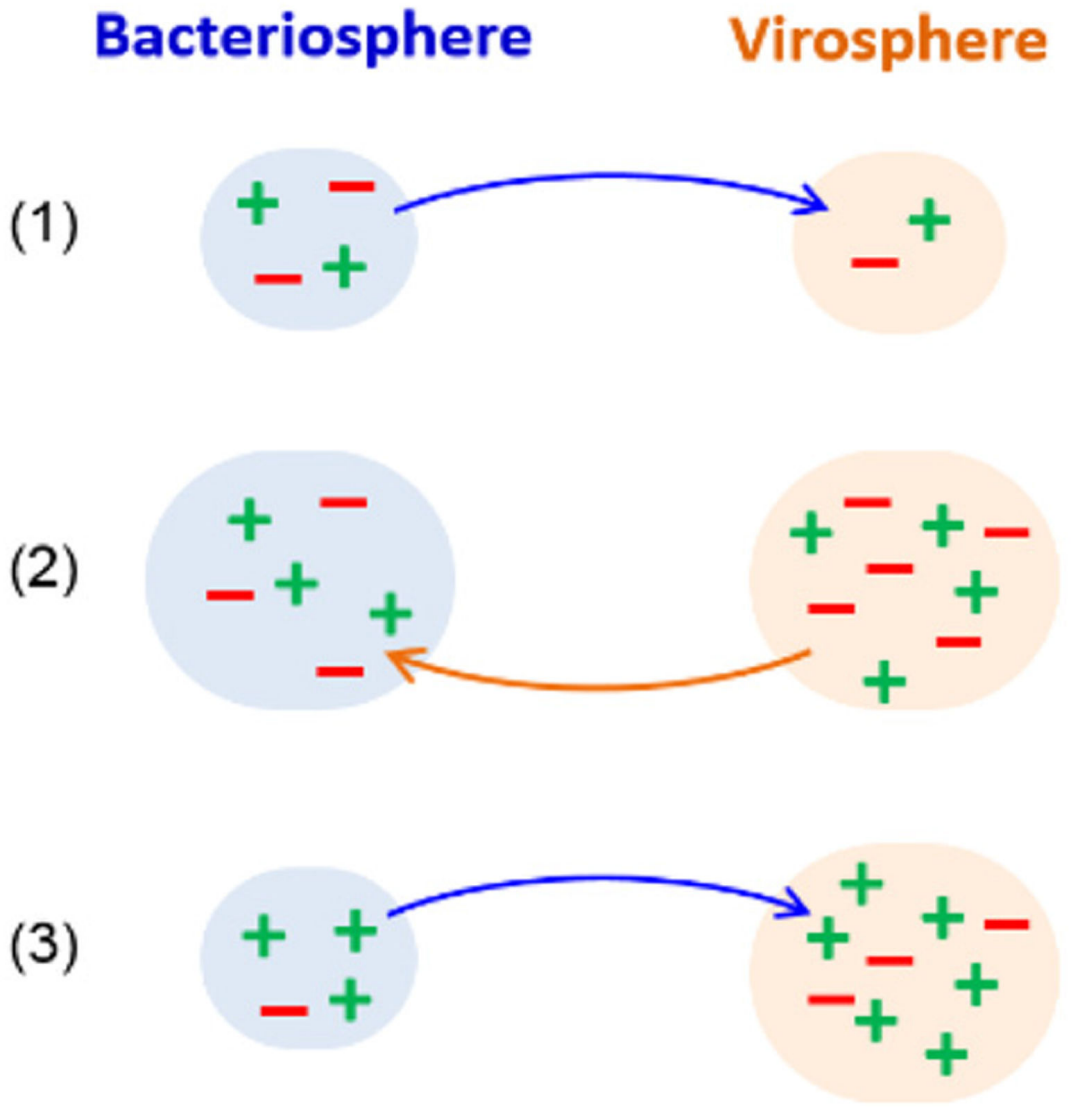
Illustration of collective coevolution between bacteria and viruses via HGT: (1) phages acquire beneficial (+) and inferior (−) genes from bacteria. (2) Phages have high mutation rate and create a rapidly evolving reservoir of genes for the host bacteria. (3) Bacteria create a slowly evolving, stable repository of beneficial genes for phages by filtering out inferior genes. The collective state results in emergent mutualism despite of individual antagonism.

What were the environmental and genetic drivers of the evolution of the high light-adapted ecotype? *Prochlorococcus* has a highly streamlined genome, and lives at low density in a nutrient-deficient environment. Thus, the required spatial adaptations were the result of novel genes that presumably were distributed through viral-mediated HGT. Consistent with this interpretation, *Prochlorococcus* does not possess standard defense mechanisms against phage attack, such as CRISPR or prophages (for restriction-modification, the situation is not clear) [61]. It seems that their principle means of defense against phage is modification of cell surface molecules that prevent phage attachment. These molecules are expressed from genes that have been rapidly modified through mutations and HGT with other bacteria phyla. These genes reside in genomic islands and constitute the majority of the genetic diversity [61]. In recent work, we have performed a calculation from a minimal stochastic model to show how HGT leads to collective coevolution of the bacteria and their phages, leading to the emergence of stratified ecotypes in the euphotic zone [122]. Through HGT from bacteria, phages acquire both beneficial and inferior genes that are responsible respectively for efficient and inefficient photosynthesis in a certain environment. Since phages have a relatively higher mutation rate, they create a rapidly evolving reservoir of genes for the host bacteria. On the other hand, bacteria with highly streamlined genomes create a slowly evolving, stable repository of beneficial genes for phages by filtering out inferior genes under selection. By carrying and transferring beneficial photosynthesis genes, there is evidence that phage improve their fitness, e.g. by optimizing their burst size, by supplementing the host cell’s metabolism [123] (figure 16). In reality most mutations are neutral or deleterious; but HGT is blind to this. Thus in HGT with host bacteria, on a fast time scale, phages evolve deleterious mutations, but can be rescued by bacteria whose genome preserves genes on a longer time scale. Eventually bacteria and phage form a collective state, enabling the rapid adaptation and range expansion to the environment nearer the ocean’s surface. This emergent mutualism occurs despite the intrinsic antagonism between bacteria and phages. In short, HGT-driven collective coevolution provides a natural unified explanation for the features of *Prochlorococcus* system, including highly streamlined small genome but huge pan-genome, lack of defense mechanisms against viral attack, niche stratification of ecotypes and phage predator carrying photosynthesis genes. It is expected that this type of mechanism can appear in other spatially-stratified systems, where genes that benefit the evolution of both host and parasite could be present.

### 12.4. Current and future challenges

A generic framework to study rapid eco-evolutionary dynamics with multi-scale feedback requires not only population dynamics and genetic evolutionary mechanisms but also the understanding of the origin of genetic variations. One related long-standing puzzle is: did the selected phenotypes already preexist or were encoded in the phenotypic variation in the ecosystem prior to the selection, or do they arise through stress-induced mutagenesis*—de novo* mutations induced by strong selection pressure at a higher rate (and perhaps at different loci) [124]? How does stress-induced mutagenesis feed back into niche construction? A crucial role is played by the genotype–phenotype map, but how is it influenced by selection in spatially and temporally varying environments?

### 12.5. Concluding remarks

We have primarily focused on the rapid evolution of microbial ecosystems, which occurs through the interplay between gene flow, spatial variation, and feedbacks between the organisms in the ecosystem and the physical characteristics of the environment. These phenomena are critical to understanding such critical issues as the emergence of antibiotic resistance [108] and the ongoing dynamics of global climate change [105-107]. Another extreme example of ecological-evolutionary feedback is the growing realization that the cancer tumor microenvironment provides a strong source of heterogeneity that underlies the rapid evolution of chemotherapy resistance [125] and the emergence of collective sensing and decision-making [126]. Theoretical modeling of these important classes of problem requires explicit handling of spatial structure and demographic fluctuations. To understand how scale-dependent ecological-evolutionary feedback drives the spatio-temporal evolution of ecosystem structure is a truly grand challenge that requires a trans-disciplinary approach to be successful.

### Acknowledgments

This material is partially supported by the National Aeronautics and Space Administration through the NASA Astrobiology Institute under Cooperative Agreement No. NNA13AA91A issued through the Science Mission Directorate.
